# Intestinal dysmotility in a zebrafish (*Danio rerio*) *shank3a;shank3b* mutant model of autism

**DOI:** 10.1186/s13229-018-0250-4

**Published:** 2019-01-31

**Authors:** David M. James, Robert A. Kozol, Yuji Kajiwara, Adam L. Wahl, Emily C. Storrs, Joseph D. Buxbaum, Mason Klein, Baharak Moshiree, Julia E. Dallman

**Affiliations:** 1 Department of Biology, University of Miami, Coral Gables, FL USA; 2 Seaver Autism Center for Research and Treatment, Department of Psychiatry, Friedman Brain Institute and Mindich Child Health and Development Institute, Icahn School of Medicine at Mount Sinai, New York, NY USA; 3 Department of Physics, University of Miami, Coral Gables, FL USA; 4 Division of Gastroenterology, Atrium Health, University of North Carolina, Charlotte, NC USA; 5 Denali Therapeutics, South San Francisco, CA USA

**Keywords:** Digestive transit, Peristaltic rate, Enteroendocrine, Phelan-McDermid syndrome

## Abstract

**Background and aims:**

Autism spectrum disorder (ASD) is currently estimated to affect more than 1% of the world population. For people with ASD, gastrointestinal (GI) distress is a commonly reported but a poorly understood co-occurring symptom. Here, we investigate the physiological basis for GI distress in ASD by studying gut function in a zebrafish model of Phelan-McDermid syndrome (PMS), a condition caused by mutations in the *SHANK3* gene.

**Methods:**

To generate a zebrafish model of PMS, we used CRISPR/Cas9 to introduce clinically related C-terminal frameshift mutations in *shank3a* and *shank3b* zebrafish paralogues (*shank3abΔC*). Because PMS is caused by *SHANK3* haploinsufficiency, we assessed the digestive tract (DT) structure and function in zebrafish *shank3abΔC*^*+/−*^ heterozygotes. Human *SHANK3* mRNA was then used to rescue DT phenotypes in larval zebrafish.

**Results:**

Significantly slower rates of DT peristaltic contractions (*p* < 0.001) with correspondingly prolonged passage time (*p* < 0.004) occurred in *shank3abΔC*^*+/−*^ mutants. Rescue injections of mRNA encoding the longest human *SHANK3* isoform into *shank3abΔC*^*+/−*^ mutants produced larvae with intestinal bulb emptying similar to wild type (WT), but still deficits in posterior intestinal motility. Serotonin-positive enteroendocrine cells (EECs) were significantly reduced in both *shank3abΔC*^*+/−*^ and *shank3abΔC*^*−/−*^ mutants (*p* < 0.05) while enteric neuron counts and overall structure of the DT epithelium, including goblet cell number, were unaffected in *shank3abΔC*^*+/−*^ larvae.

**Conclusions:**

Our data and rescue experiments support mutations in *SHANK3* as causal for GI transit and motility abnormalities. Reductions in serotonin-positive EECs and serotonin-filled ENS boutons suggest an endocrine/neural component to this dysmotility. This is the first study to date demonstrating DT dysmotility in a zebrafish single gene mutant model of ASD.

**Electronic supplementary material:**

The online version of this article (10.1186/s13229-018-0250-4) contains supplementary material, which is available to authorized users.

## Background

Autism spectrum disorder (ASD) is estimated to impact more than 1% of the population and is etiologically and clinically heterogeneous [1, 2]. While ASD diagnoses are based upon deficits in social communication and the presence of repetitive behaviors and/or restrictive interests, co-occurring symptoms (comorbidities) are also common. Here, we focus on gastrointestinal (GI) distress, one of the more frequent comorbidities experienced by individuals with ASD [3, 4]. Despite the negative impacts of GI distress on daily life, ASD-associated GI symptoms are poorly understood [3, 5]. Consistent with prospective findings, clinical reports from monogenic causes of ASD regularly document GI distress [3, 6–8]. Our work focuses on a monogenic form of ASD, Phelan-McDermid syndrome, that is caused by mutations that disrupt one copy of the *SHANK3* gene resulting in *SHANK3* haploinsufficiency [9, 10]. In individuals with PMS, GI distress is characterized by reflux, cyclical vomiting, diarrhea, and/or constipation [11, 12]. To investigate the biological mechanisms underlying GI distress in PMS and ASD, we have generated a zebrafish *shank3* mutant model.

The majority of SHANK3 loss-of-function animal models are mammalian and have provided great insight into neural mechanisms related to social and motor behaviors characteristic of ASD [13]. SHANK3 is known to act as a synaptic scaffolding protein in the central nervous system (CNS) where it helps to regulate synaptic development, glutamatergic receptor signaling, actin polymerization, and dendritic spine formation [14–19]. In addition, SHANK3 is also expressed at early developmental stages prior to synapse formation [20, 21], as well as in enterocytes and nitrergic neurons of the enteric nervous system (ENS) [22–24], and has been shown to have important interactions with the Wnt signaling pathway [25]. Studies suggest that SHANK3 may also play important GI-related roles in host/symbiont interactions and Zn metabolism [22, 26–28] and intestinal barrier function [29]. Studies to explore roles for SHANK3 in relation to GI dysfunction, however, are limited.

To understand the etiology of ASD symptoms, zebrafish is a powerful model system [30–32]. Genetically and physiologically similar to humans and mammalian models, zebrafish provide a complementary model system with accessible developmental stages that are transparent, allowing physiological assessment in vivo [31, 33–35]. Additionally, zebrafish and human digestive tracts are largely conserved, with similar hormonal regulation, morphology, cell types, and physiology, albeit simplified in zebrafish [8, 36–40]. For example, in both zebrafish and mammals, digestion rate adapts to the size of the meal [39]; also in both, serotonin, acetylcholine, motilin, and ghrelin increase DT motility [39, 41, 42] while vasoactive intestinal peptide, pituitary adenylate cyclase-activating peptide, and nitric oxide decrease DT motility [41, 43]. Like mammals, the zebrafish DT tract can be divided into sections distinguished by differences in cell type and function: digestive secretions are enriched anteriorly where both nutrient absorption and tissue folding are the greatest, while posterior regions are largely devoid of folding and cell types are largely specialized for water absorption [44, 45]. Additionally, the ENS and key DT regulatory brain regions such as the hypothalamus, secondary gustatory nuclei, vagal motor nucleus, sensory nodose ganglia, and spinal dorsal root ganglia are conserved in zebrafish [32, 40, 46, 47]. Experiments that have used zebrafish to investigate GI dysfunction in Hirschsprung’s and chronic intestinal pseudo-obstruction diseases have found that disrupting conserved zebrafish genes linked to these diseases disrupts both GI motility and ENS development [48, 49], mirroring human symptoms and supporting translatability of the zebrafish model system.

Here, we generate a zebrafish *shank3* mutant model to investigate a causal link between *shank3* loss-of-function mutations and DT dysfunction. As a result of a genome duplication approximately 300 mya [50], the *shank3* gene, along with many other synaptic genes, is duplicated in teleost fishes [51, 52], yielding *shank3a* and *shank3b* gene paralogues. Therefore, to model human *SHANK3* mutations linked to PMS [53], we used CRISPR/Cas9 to generate frame-shift mutations in the C terminal, proline-rich domains of both zebrafish *shank3a* (chromosome 18) and *shank3b* (chromosome 4) (referred to as *shank3abΔC*).

## Methods

Additional materials and methods on histology, statistics, and the Igor Pro code can be found in Additional file 1

### Fish maintenance and husbandry

Zebrafish were housed in the University of Miami zebrafish core facility. Both adult and larval zebrafish were maintained at 28 °C in system water and exposed to a 14:10 h circadian light:dark cycle. Zebrafish lines used in this study include AB wild type (WT) and transgenic lines la118Tg:Tg(*aldoca:gap43-Venus*) [54] and Tg(*vglut2a:DsRed*) [55]. To generate *shank3abΔC*^*+/−*^ animals, WT females were crossed with *shank3abΔC*^*−/−*^ males. ZFIN zebrafish gene and protein nomenclature conventions were followed.

### Single-stranded guide RNA (sgRNAs) design

Site-specific CRISPR-Cas9 sgRNAs were generated using the online software CHOPCHOP [56]. CHOPCHOP-generated guides were chosen based on location within the gene and minimal predicted off-target sites. Guides were designed to target the C-terminal proline-rich domain of *shank3a* and *shank3b* (Additional file 1: Table S1). To produce sgRNA templates for in vitro transcription, phage T7 promoter-adapted sgRNA oligonucleotides were annealed to a universal tracRNA oligonucleotide with complementary overhangs [57]. Each sgRNA template reaction contained: 1× transcription buffer, 1 μM site-specific oligo, 1 μM tracRNA oligo, 500 nM dNTPs, 0.5 U Phusion high-fidelity DNA polymerase (New England Biolabs, NEB; Ipswich, MA), and nuclease-free H_2_O to 10 μL. Reaction mixtures were heated to 95 °C for 1 min, cooled to 52 °C (0.1 °C/sec), and extended at 72 °C for 10 min. Templates were then used to transcribe single-stranded sgRNAs with a T7 MEGAscript in vitro transcription kit (Ambion; Foster City, CA) following the manufacturer’s protocol. Transcribed sgRNAs were then cleaned using ammonium acetate/ethanol precipitation and quantified by comparison to an RNA sample of known concentration on an agarose gel.

### CRISPR-Cas 9 mutagenesis and allele screening

In vitro transcribed sgRNA and Cas 9 protein (PNA Bio, Newbury Park, CA) were mixed and incubated at 37 °C for 5 min. A micro-injector was then used to inject 400:100 pg of sgRNA:Cas 9 into the cell of one-cell stage zebrafish embryos. Injected embryos were allowed to develop for 24 h, and genomic DNA (gDNA) was extracted to screen for mutations. Genes were targeted singly for either *shank3aΔC* or *shank3bΔC* then F1 mutants were out-crossed to obtain F2 *shank3abΔC* heterozygotes. These F2 mutants were then outcrossed to la118Tg:Tg(*aldoca:gap43-Venus*) and Tg(*vglut2a:DsRed*) and finally backcrossed to generate F4 and F5 *shank3abΔC* homozygous mutants. To assay mutations, polymerase chain reaction (PCR) primers were designed to flank the sgRNA target site (Additional file 1: Table S2). Each PCR reaction mixture contained: 1× GOTaq (Promega, Madison, WI), 50 nM forward and reverse primer, 1 μL gDNA, and nuclease-free H_2_O to 10 μL. PCR products were then sequenced on an AB3130 Sanger sequencer with each sequence PCR mixture containing: 1× BigDye Buffer, 50 nM forward primer, 0.3× 3.1 BigDye mixture, and 1 μL purified PCR product. Identified mutations were then analyzed for unique restriction digest sites that could be used for genotyping (Additional file 1: Figure S1). Post-experimental analysis genotyping using PCR and restriction enzyme digests (Additional file 1: Figure S1) allowed us to conduct experiments blind to genotype.

### Western

One hundred milligrams of whole fish brains dissected and pooled from adult *shank3ab* ^+/+^ (*n* = 8) and *shank3abΔC* ^*−/−*^ mutants (*n* = 8) were used to prepare the postsynaptic density (PSD) fractions following the previously published method [16]. Mouse cortices from *Shank3*^+/+^ or *Shank3*^−/−^, lacking all Shank3 isoforms, (unpublished) were processed in parallel. Briefly, brains were homogenized in HEPES buffer containing 0.32 M sucrose and protease inhibitors followed by two rounds of low-speed centrifugation (700 g) to remove nuclei. Supernatants were centrifuged at 18,000*g* to precipitate crude synaptosomal fraction, which were further homogenized in hypotonic HEPES buffer to eliminate synaptic vesicles. Finally, PSD fractions were obtained by four rounds of washing with HEPES buffer containing 0.5% Triton X-100 and high-speed centrifugation (180,000*g*) and dissolved in HEPES buffer containing 1.8% SDS and 0.85 M urea. PSD lysates were run on SDS-PAGE gel and transferred to PVDF membrane for immunoblot analysis using antibodies against Shank3 (1:100; sc-30,193, Santa Cruz Biotechnology, CA, raised against amino acids 1431–1590 mapping near the C-terminus of isoform 2 of human SHANK3), Tubulin (1:500, ab18207, Abcam), and Actin (1:500, A2066, Sigma).

### Cryosectioning and immunohistochemistry

Cryosectioning and antibody staining were performed using previously published methods [58]. Anti-PSD-95 (1:500; Abcam; Cambridge, UK; ab-18,258) and anti-SHANK3 (1:200; sc-30193, Santa Cruz Biotechnology, CA) were used as primary antibodies with secondary antibodies conjugated to Alexa Fluor 568 (Abcam, ab175472) and Alexa Fluor 488 (Thermo Fisher Scientific, R37118), respectively. Images were collected on a SP5 confocal microscope (Leica; Wetzlar, DE) using 40× and 63× oil immersion lenses.

### Peristalsis videography and analysis

To quantify DT motility, we captured videos of gut peristalsis in intact transparent 7-day-old zebrafish larvae after feeding with a chicken egg yolk emulsion. The yolk emulsion promoted more uniform feeding across the population, making screening easier. To create the emulsion, 1 ml of chicken egg yolk was rapidly and repeatedly pipetted into 1 ml of system water; several drops of the emulsion were then added to a 10 cm petri dish housing 50–100 larva. Both WT controls and *shank3abΔC* mutant larvae were exposed to the yolk mixture for the same time period (60–90 min). To avoid requiring anesthetic, videos were acquired by embedding single larvae oriented on the sagittal plane in low melt point agarose (LMA; Thermo Fisher, BP165-25) on a glass-bottom 35 mm petri dish. Videos were recorded at 1 frame/second for a total of 10 min on a Canon EOS 5D Mark III; larvae were then processed for genotyping. Videos were compiled blind to genotype, and run through a custom script in Igor Pro. This script allows a user to demarcate a specific area of analysis, or AOA (Fig. 3a, red lines), and determine the number of regions to evaluate along the area of analysis (Fig. 3a, cyan boxes) and the amount of raster averaging for each AOA. Post-analysis, the program produces individual peristaltic periodicity per point of evaluation and overall averages of peristaltic periodicity (see Additional file 1). These outputs were manually annotated to check for artifacts due to muscle twitches that were easy to distinguish from DT peristalsis due to the much faster timescale.

### DT transit

Seven dpf *shank3abΔC* ^*+/−*^ mutant and WT larvae were fed a mixture of 6 μm fluorescent beads (Polysciences 17156, fluoresbrite beads packaged as 2.5% aqueous suspension) and chicken egg yolk emulsion for 1.5 h. Approximately 10 μl of fluorescent bead solution in 2 ml of egg yolk emulsion was added to the 10 cm petri dish containing the larvae in approximately 20 ml system water. After screening for individuals who had filled their intestinal bulb with the yolk-bead suspension, larvae were embedded in LMA on a 35-mm, glass-bottom petri dish, and a series of pictures of each larva were taken with a Zeiss Axiocam on a stereoscope V20 at 3, 6, 12, and 24 h. Regions of analysis were the pharynx, intestinal bulb, upper intestinal tract, lower intestinal tract, and expelled. This procedure was adapted from [59]. Calculations were made blind to genotype. Using FIJI, the total area encompassed by the fluorescent beads was determined, and percentages of this total were calculated per DT region. Averages for 3, 6, 12, and 24 h time periods were compiled for both WT and *shank3abΔC* ^*+/−*^ larvae. Supplementary movie figures (*shank3abΔC* ^*+/−*^, Additional file 2: Movie S1; and WT, Additional file 3: Movie S2) were processed as described above. Individuals were screened for high quantities of fluorescent intestinal content and were embedded as described. Images were acquired using a Zeiss Axio Imager.Z2 paired with a Zeiss AxioCam MRm Rev3 camera running Zeiss Zen Blue software. Images were acquired every 10 s for 6 h. Image acquisition began late morning and was concluded before 5 pm; as such, the room lights were left on during the duration of the recording process to prevent any confounding effects with shifting circadian rhythm.


Additional file 2: Movie S1. Playback at 5 frames per second, each frame represents 1 z-stack capture per minute. This movie shows the abnormal mutant peristaltic process in a 7 dpf *shank3abΔC*^*+/-*^ zebrafish. The fluorescent microspheres can be seen near the intestinal bulb-upper intestine junction; this was a common catch point and the mutant model seems to have the greatest difficultly moving food particles out of the intestinal bulb. Post processing was done to reduce size, change orientation of images, and speed playback up. (MP4 16987 kb)



Additional file 3: Movie S2. Playback at 5 frames per second, each frame represents 1 z-stack capture per minute. This movie shows “normal” wild type peristaltic movements of a 7 dpf zebrafish, highlighting the intestinal region starting at the intestinal bulb-upper intestine junction. Fluorescent microspheres can be seen moving posteriorly down the digestive tract. Post processing was done to reduce size, change orientation of images, and speed playback up. (MP4 23290 kb)


### Rescue experiments

Fertilized *shank3abΔC* ^*+/−*^ embryos were injected with a full-length (designated 5t) human *SHANK3* mRNA, or a short isoform (designated 32t, Additional file 1: Table S9) [20]. These fish were allowed to grow to 7 dpf before being analyzed with the microsphere transit assay listed above. Additionally, control injections of loading dye and non-injected WT and *shank3abΔC* ^*+/−*^ fish were analyzed.

### Quantification of gut ENS and enteroendocrine cells

Seven dpf WT and mutant larvae were incubated on ice for 30 min before being fixed in 4% Formaldehyde (Pierce^TM^, Thermo Fisher Scientific, 28906)-1× phosphate buffer solution/0.25% Triton X-100 (1× PBSTx) for 1 h at room temperature. Whole-mount antibody staining and gut dissections were carried out according to previously published methods [60]. Anti-HuC/HuD (1:1000; Invitrogen; A21271) and anti-5-HT (1:1000; serotonin; Immunostar, New Richmond, WI; 20,080) were used as primary antibodies with secondary antibodies conjugated to Alexa Fluor 568 (1:1000; Abcam, ab175472) and Alexa Fluor 633 (1:1000; Thermo Fisher Scientific, A-31575). To sample the anterior and posterior intestinal tract, a 200 × 200-μm field was captured 200 μm from the intestinal bulb (anterior) and 200 μm from the anus (posterior) as in [61]. Images were captured using a 20× dry objective and analyzed using the cell counter application in Fiji.

## Results

### Generating zebrafish *shank3abΔC* loss-of-function mutant models of Phelan-McDermid syndrome

**Fig. 1:**
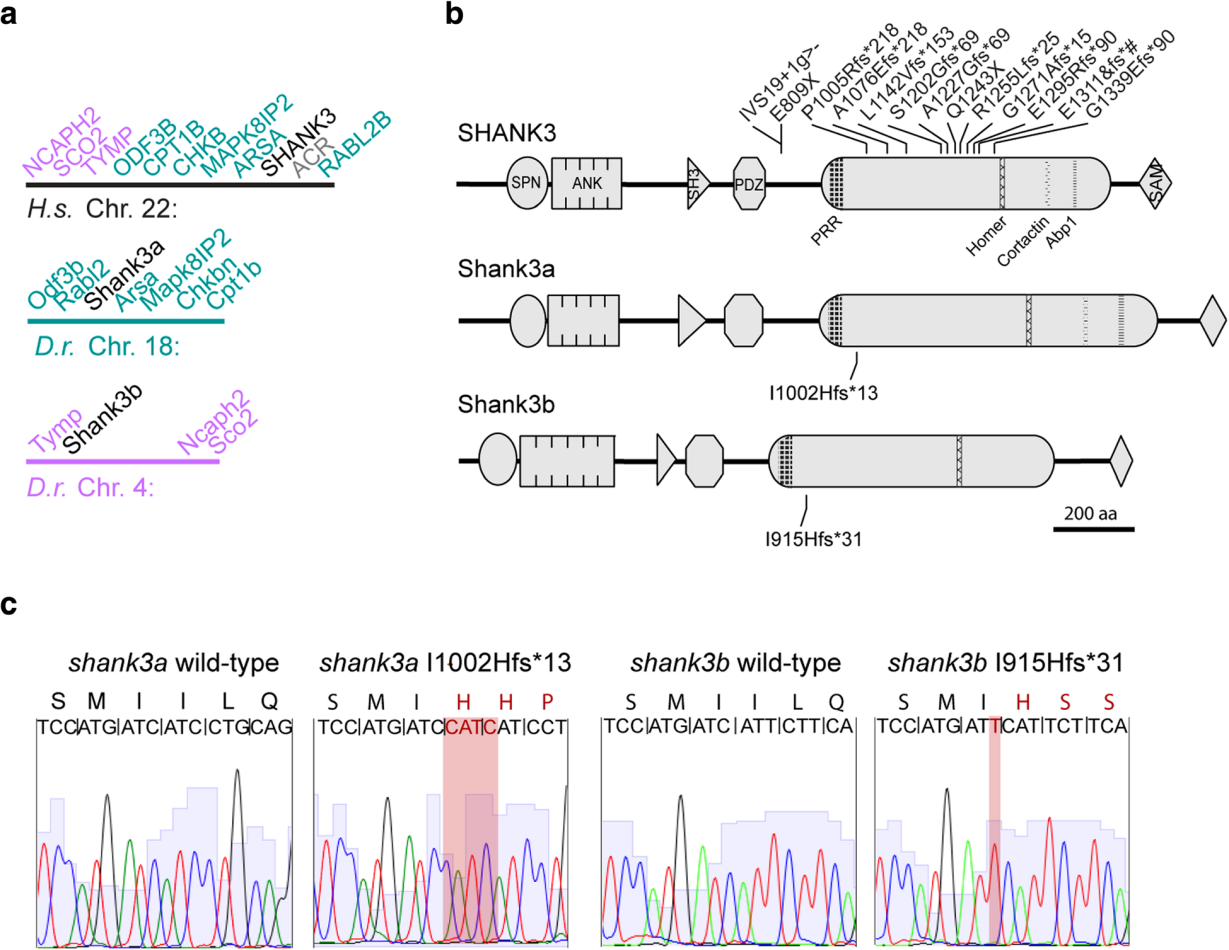
*SHANK3* orthologues are conserved in zebrafish and CRISPR/Cas9-induced frameshift mutations are similar to those found in people with Phelan-McDermid syndrome. **a** *shank3a* on chromosome 18 (teal) and *shank3b* on chromosome 4 (purple) retain synteny with different genes surrounding *SHANK3* on chromosome 22 in humans. **b** Shank3a and Shank3b retain protein binding domains found in humans including Shank/ProSAP N-terminal (SPN), ankyrin repeats, SRC Homology 3 (SH3), postsynaptic density protein/disc large tumor suppressor/zonula occludens-1 protein (PDZ), proline-rich region (PRR), Homer-interaction, cortactin-interaction, actin-binding protein 1 (Abp1), and sterile alpha motif (SAM). Individuals with *SHANK3* mutations show a C-terminal bias affecting the largest coding exon preceding the SAM-domain encoding exon (gray cylinder). All indicated mutations show frameshift mutations from people with Phelan-McDermid syndrome above and the two CRISPR generated mutations in zebrafish below. **c** Raw sequence traces show insertion mutations highlighted in red with corresponding changes in downstream amino acids. Mutations were named according to changes in amino acids (e.g., *shank3b* isoleucine 915 changed to histidine with 31 frame-shifted amino acids terminating in a stop codon)

Zebrafish *shank3a* on chromosome 18 shares human SHANK3 synteny, surrounded by six of the seven genes that neighbor human *SHANK3*, while *shank3b* has reduced synteny, flanked by three of the genes that are more distant from human *SHANK3* (Fig. 1a). Moreover, while zebrafish Shank3a retains all known protein binding domains, Shank3b has an additional ankyrin repeat and lacks cortactin and Abp1 interaction domains (Fig. 1b). CRISPR-Cas9/sgRNA injections produced frameshift mutations in *shank3a* and *shank3b*. Both sgRNAs targeted the largest exon in *shank3* that encodes a large C-terminal region containing several protein interaction domains. Similar human frameshift mutations in the *SHANK3* C-terminal domain have been associated with ASD [53, 62, 63]. Frameshift mutations were induced in both *shank3a* and *shank3b* genes, and alleles were selected to minimize the number of amino acids between frameshift and stop codon (Fig. 1c). In *shank3a*, a four-base pair insertion converted an isoleucine into a histidine and introduced thirteen amino acids before the stop codon. In *shank3b*, a single-base pair insertion converted an isoleucine into a histidine and introduced thirty-one amino acids before the stop codon.

**Fig. 2:**
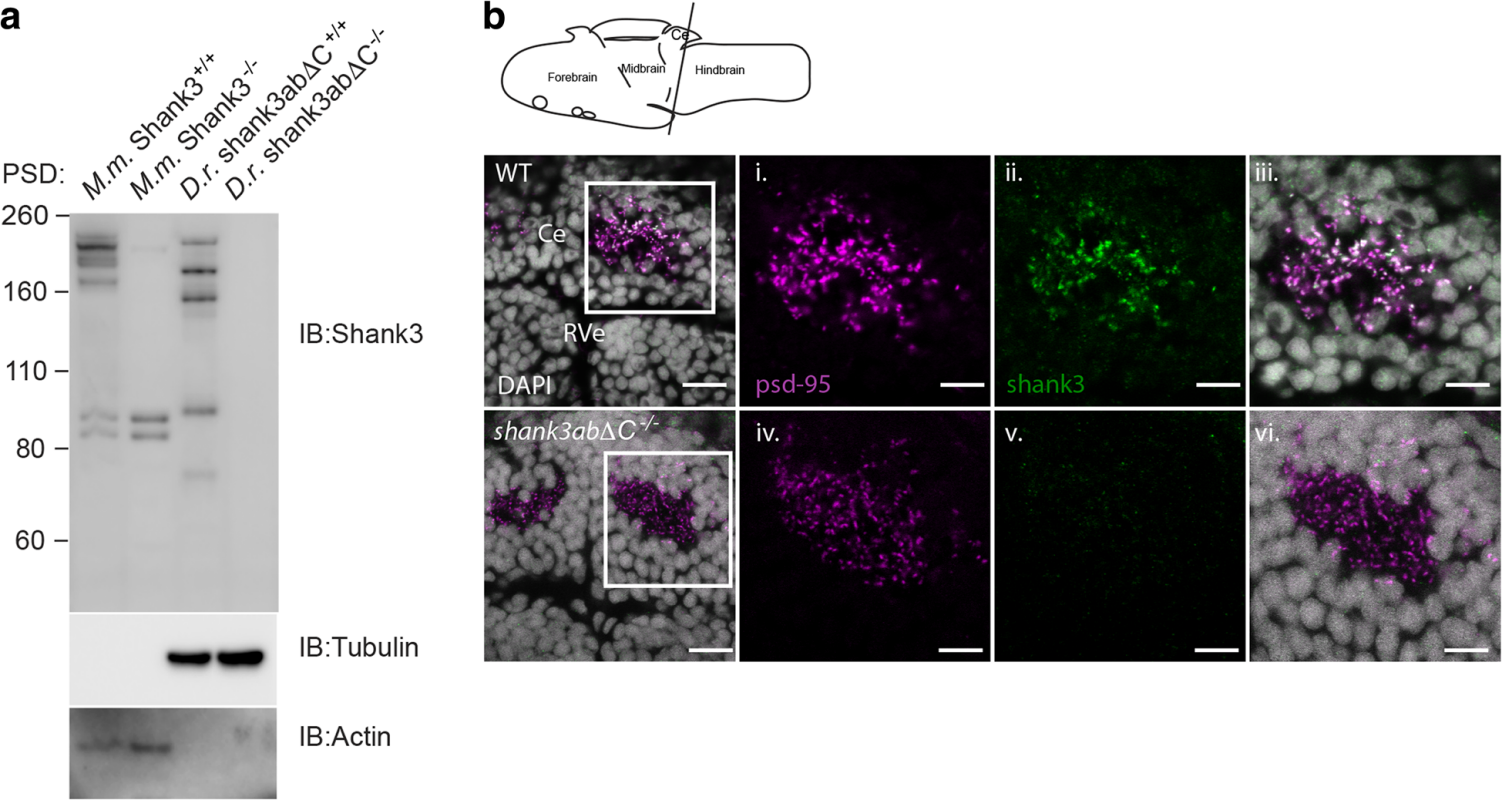
Zebrafish Shank3ab protein shares complex isoform expression with mammals and shows expression in the cerebellum. **a** Shank3 western blots of postsynaptic densities (PSD) isolated from mouse (*M.m*) and zebrafish (*D.r.*) show similar complex isoform expression. Molecular marker weight is expressed in kilodaltons. Tubulin and actin expression were used as loading controls for zebrafish and mouse immunoblots, respectively. Loss of Shank3ab staining in IHC and the western blot supports the immunoreactive specificity of Shank3 antibody. **b** Shank3ab protein is expressed as distinct overlapping puncta with PSD-95 in the cerebellum from larvae 6 days post-fertilization. Transverse cerebellar sections (see diagram) with enlarged insets show neuropil area dorsal to the rhombencephalic ventricle (RVe). Scale bar represents 10 μm for the first column and 5 μm for the insets (i–vi)

A western blot of protein samples enriched for membrane fractions including post-synaptic densities (PSDs) was then used to determine whether the mouse Shank3 antibody was cross-reactive in zebrafish and to characterize the isoform complexity of zebrafish Shank3ab. Zebrafish protein extracts from adult brain showed multiple isoforms as indicated by the five bands of ranging from 75 to 250 kD in WT. For comparison, PSDs isolated from WT and *Shank3*^*−/−*^ mouse brains showed similar Shank3 isoform complexity (Fig. 2a). By contrast, PSD fractions from zebrafish mutant for both *shank3a* and *shank3b* (*shank3abΔC* ^−/−^) showed no visible bands confirming the specificity of the mouse Shank3 antibody for zebrafish Shank3ab.

To test for localization of Shank3ab protein in 6 days post-fertilization (dpf) zebrafish brain tissue, we stained fresh frozen sections from both *shank3abΔC* ^−/−^ and WT larvae. Sectioned tissue was co-labeled with PSD-95 antibody because Shank3 is known to co-localize with PSD-95 at mammalian glutamatergic post-synapses [64]. As in mammals, we found that PSD-95 and Shank3 expression co-localized in post-synaptic puncta of WT larvae (Fig. 2b). These puncta were found in the cerebellum that most likely label glutamatergic granule cell synapses. By contrast, Shank3ab expression was absent in *shank3abΔC* ^−/−^ mutants despite robust PSD-95 staining.

### DT peristalsis frequency is reduced in *shank3abΔC* larvae

**Fig. 3:**
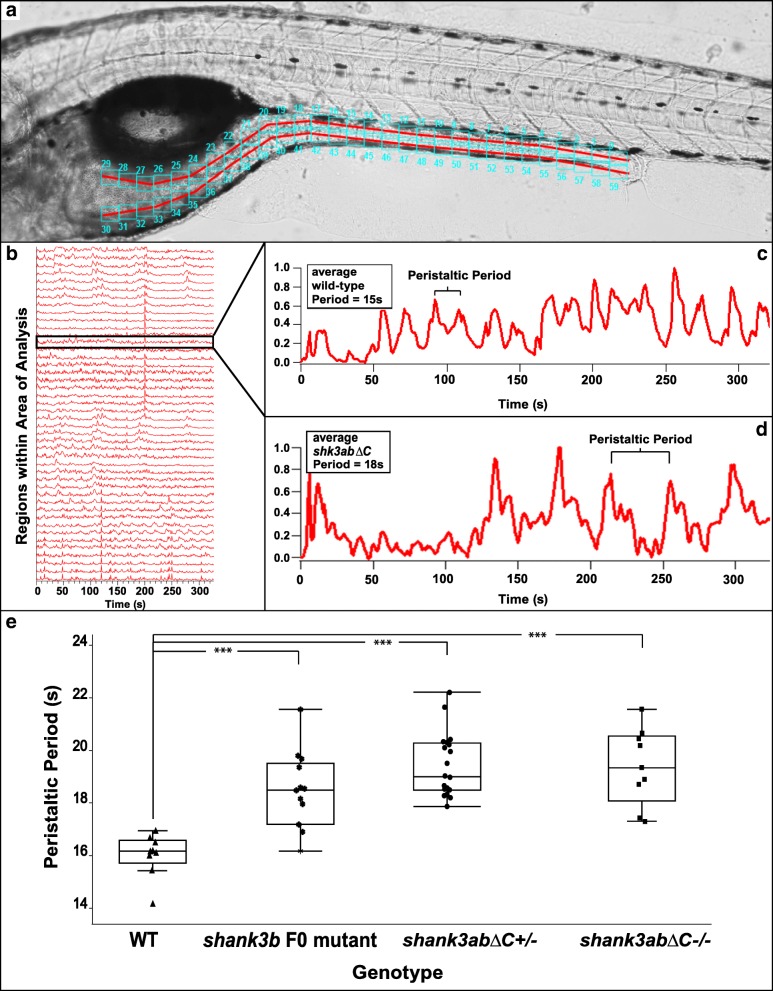
*shank3abΔC*^−/−^ and *shank3abΔC*^+/−^ larvae have reduced peristaltic frequencies and DT motility compared to WT. **a** Representative image of a zebrafish embedded for analysis, with the area of analysis (AOA) denoted by the red lines and the regions of evaluation denoted by the cyan boxes (1–60). The AOA was manually drawn in, and the program automatically fills a set number of recording regions along the AOA. For our work, this number was set at 30 dorsal and 30 ventral, but the number and size of the boxes can be adjusted if necessary. **b** Representative WT data output, showing output from all regions of evaluation within the AOA; as the peristaltic contraction moves down the DT tract, the Igor Pro program registers the change in pixel intensity. **c** Representative image of an individual analysis readout for a WT larva, with the change in pixel intensity (*y*-axis) measured as time progresses (*x*-axis). Igor Pro can separate individual regions of evaluation within the AOA and give data on peristaltic periodicity in a specific region, allowing users to get overall averages throughout multiple regions within the AOA. **d** Representative image of an individual analysis readout for a *shank3abΔC* ^+/−^ larvae, note the larger space between peaks, indicating reduced peristaltic frequencies. **e** WT larvae are shown to have peristaltic rates significantly (*p* < 0.0001) more frequent than that of *shank3abΔC* mutants. F_0_ shank3b larvae are significantly slower than WT, while F_0_, *shank3abΔC* ^+/−^ and *ΔC* ^−/−^ show no significant difference between each other. Each point represents an average frequency from a 7-day-old larva

To test whether *shank3abΔC* mutations impact DT function, we compared peristaltic rates in WT and different combinations of *shank3abΔC* mutant alleles in 7 dpf larvae. After feeding, larvae were filmed for a period of 10 min to capture the muscular contractions moving along the DT tract. To determine peristalsis frequency, a custom script in Igor Pro quantifies pixel intensity changes to produce individual plots for regions along the DT (Fig. 3a, cyan boxes). These data show that *shank3abΔC*^*+/−*^ larvae have significantly less frequent peristaltic contractions compared to WT larvae (Fig. 3d, WT *n* = 9, *shank3abΔC*^*+/−*^*n* = 29, *p* < 0.001). Peristaltic contractions are shown for the intestinal bulb, as this region is the most responsive to a feeding event. It should be noted, however, that this pattern of significantly reduced motility was also seen in upper and lower intestines (Additional file 1: Table S7). In addition to *shank3abΔC*^*+/−*^ larvae, this dysmotility phenotype was also seen in both CRISPR/Cas9/*shank3b* sgRNA injected embryos (F_0_ generation; *n* = 13; *p* = 0.0001) and *shank3abΔC*^*−/−*^ larvae (*n* = 9, *p* < 0.0001) compared to WT (similar results were found in the CRISPR/Cas9/*shank3a* sgRNA injected embryos, but were not recorded with sufficient *n* to include in the data). Differences between heterozygous and homozygous *shank3abΔC* mutants were not found to be significant (*p* = 0.978), suggesting a maximum level of DT effect in *shank3abΔC* ^*+/−*^ genotypes. Our subsequent analyses therefore focus on this *shank3abΔC* ^*+/−*^ phenotype that best models human *SHANK3* haploinsufficiency.

### DT transit time is prolonged in *shank3abΔC*^+/−^ larvae

**Fig. 4:**
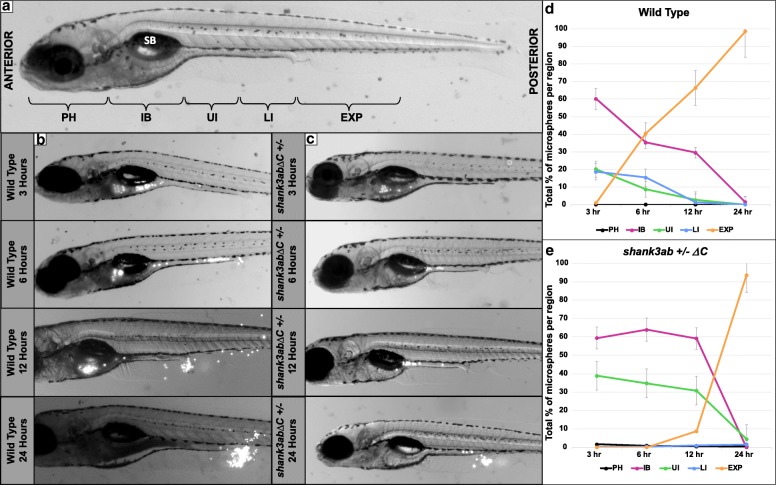
DT transit time of fluorescent beads is prolonged in *shank3abΔC*^*+/−*^ mutants compared to WT larvae. **a** Regions of the DT analyzed for fluorescent bead occupancy are indicated on a 7-day-old zebrafish larva. Beads were tracked as they passed through five key regions: PH (pharynx), IB (intestinal bulb), UI (upper intestine), LI (lower intestine), and EX (expelled). The swim bladder (SB) is labeled for reference. **b** Images of a representative WT fish at 3, 6, 12, and 24 h post-feed. Beads appear white and can be seen throughout the DT and collecting near the anus post-expulsion. **c** Images of a representative *shank3abΔC* ^+/−^ larva at 3, 6, 12, and 24 h post-feed. **d** Graph of bead distribution per time point for WT fish. Each point represents the average +/− standard error of the percentage of total beads held by each region (PH, IB, UI, LI, or EXP) over time (*n* = 13). **e** Graph of *shank3abΔC* ^+/−^ mutant average +/− standard error bead distribution over time (*n* = 15)

Having established a DT motility phenotype associated with *shank3abΔC* ^*+/−*^ mutants, we next tested whether the reduced peristaltic frequencies translated into prolonged DT transit times by feeding larvae fluorescent 6 μm microspheres in an emulsion of egg yolk and timing microsphere transit through the DT. Both WT and *shank3abΔC*^*+/−*^ larvae were embedded in 1% LMA, immersed in system water, and photographed at 3, 6, 12, and 24 h. Transit differed between WT and *shank3abΔC* ^*+/−*^ mutants, both in terms of overall rate and the amount of time spent in the upper DT (Fig. 4; Additional file 1: Tables S3–S6). To quantify these differences, we compared the proportions of fluorescent microspheres in DT regions across time and genotypes. After permutation (Additional file 1: Table S8), a repeated measures ANOVA found significant interactions in the percentage of microspheres between genotype (*shank3abΔC*^*+/−*^, *n* = 15 and WT, *n* = 13) and time (*p* < 0.004). When analyzing the rate of bead expulsion specifically (analogous to completed DT transit), significant differences were found between WT and *shank3abΔC* ^*+/−*^ larvae at 6 h and 12 h transit times (*p* < 0.006), but not the 24 h time point (*p* = 0.330). This is indicative of a severely delayed but not entirely obstructed transit. Most WT larvae had passed a large portion of the consumed microspheres by 6 h and had passed the remainder between 12 and 24 h post consumption (Fig. 4b). Comparatively, *shank3abΔC* ^*+/−*^ larvae took longer than 12 h to begin passing the microspheres and some individuals had not passed the remainder even after 24 h post consumption (Fig. 4c). Of particular interest is the amount of “sloshing” that occurred in *shank3abΔC* ^*+/−*^ larvae, where the microspheres would repeatedly move anteriorly and posteriorly between the intestinal bulb and upper-intestine. In *shank3abΔC* ^*+/−*^ larvae, the passage of particles seemed to get delayed in two key areas: the intestinal bulb to upper-intestine transition and near the anus (Additional file 2, *shank3ab* mutant). This intestinal bulb to upper-intestine transition marks a key region where both anterograde and retrograde peristaltic propulsion transitions to just retrograde propulsion [36]. WT larvae displayed no sloshing and passed the microspheres in a clear anterior to posterior direction; WT similarly lacked the delay in the intestinal bulb to upper-intestine transition that was seen in *shank3abΔC* ^*+/−*^ larvae.

### Partial rescue of *shank3abΔC*^+/−^ transit time with human *SHANK3*

**Fig. 5:**
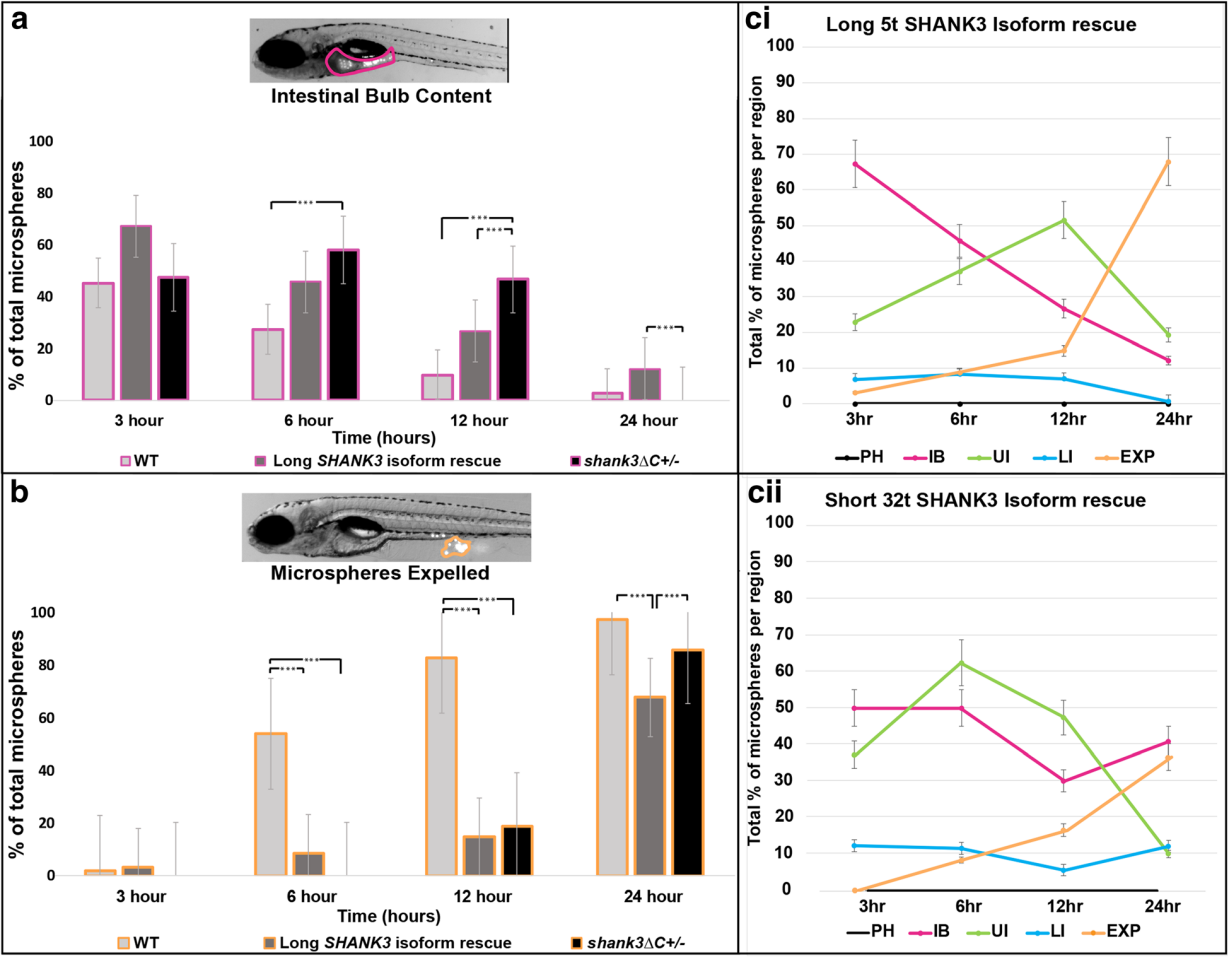
Full-length human *SHANK3* mRNA partially rescues transit time in *shank3abΔC* ^+/−^ larvae. **a** When measuring the total percentage of fluorescent beads within the intestinal bulb (highlighted region of analysis for in magenta), total content of the long isoform (5t) rescue (dark gray) decreases at a rate quicker than the unrescued *shank3abΔC* ^+/−^ (black) but not as quickly as the WT (light gray). Significant differences of intestinal bulb emptying (total intestinal bulb content) were found between the long isoform rescued and unrescued *shank3abΔC* ^*+/−*^ larvae at 12 h (*p* < 0.006) and 24 h (*p* < 0.03), and between WT and unrescued *shank3abΔC* ^+/−^ at 6 h (*p* < 0.02) and 12 h (*p* < 0.01). **b** Fluorescent microbead expulsion (analogous to completed digestion; highlighted region of analysis in orange) was not rescued, however, and the long isoform rescued larvae showed expulsion rates closer to *shank3abΔC* ^+/−^ than that of WT. Significant differences were found at 6 h between WT, the long isoform rescue, and unrescued *shank3abΔC* ^+/−^ (*p* < 0.006), at 12 h between WT and unrescued *shank3abΔC* ^+/−^ (*p* < 0.001), WT and the long isoform rescue (*p* < 0.001), and at 24 h between unrescued *shank3abΔC* ^+/−^ and the long isoform rescue larvae (*p* < 0.01), and WT and the long isoform rescue larvae (*p* < 0.005). **ci** Graph of bead distribution per time period for the long isoform (5t) rescue larvae (*n* = 19) and **cii** short isoform (32t) rescue larvae (*n* = 6). Each point represents the average +/− standard error percentage of total beads held by each region

To test whether providing human *SHANK3* mRNA to *shank3abΔC* ^*+/−*^ zebrafish embryos is sufficient to rescue the DT dysmotility phenotype, we injected mRNA encoding either the longest human *SHANK3* isoform that includes all SHANK3 protein domains (5t, *n* = 19) or a shorter human *SHANK3* isoform that includes only the C-terminal proline-rich and SAM domains (32t, *n* = 6) into fertilized eggs from *shank3abΔC* ^*+/−*^ mutants. After injection, larvae were reared to 7 dpf and were put through the fluorescent microsphere digestive transit assay. While injecting *shank3abΔC* ^*+/−*^ larvae with the short 32t *SHANK3* isoform further slowed mutant transit times (Fig. 5cii), injecting the long 5t *SHANK3* isoform partially rescued mutant transit times (Fig. 5ci); microspheres moved in a clear anterior-posterior direction without any sloshing or pause at the intestinal bulb to upper-intestine transition. The rescue did not return the WT phenotype entirely; while the progression of microspheres was more consistent, the transit rates in rescued *shank3abΔC* ^*+/−*^ mutants resembled a mid-point between unrescued *shank3abΔC* ^*+/−*^ and WT larvae (Fig. 5a). By contrast to the partial rescue in the upper intestine, transit rates in the lower intestine were not rescued (Fig. 5b). Therefore, it is likely that RNA rescue was associated with overexpression phenotypes in addition to the partial functional rescue seen with injection of the longest *SHANK3* isoform.

### Larval DT morphology

**Fig. 6:**
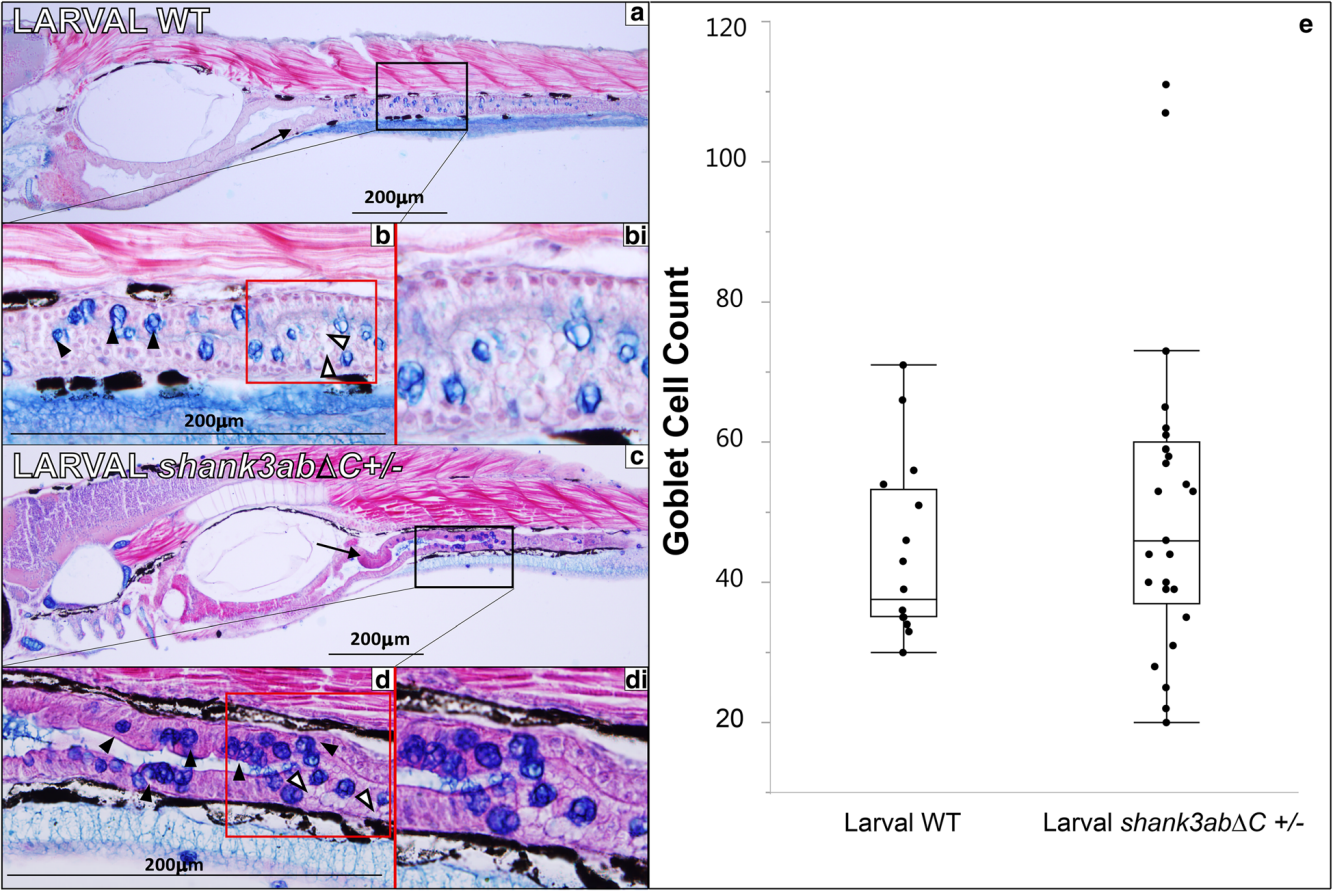
Histology of larval WT and *shank3abΔC*^+/−^ mutants (*n* = 25 for *shank3abΔC* ^*+/−*^*n* = 15 for WT). Black boxes in **a** and **c** indicate regions shown at 40× magnification, while red boxes in **b** and **d** indicate regions of magnified insets in **bi** and **di**. **a** 10× magnification of longitudinal sections through 7 dpf WT fish (anterior left to posterior right); sections were stained with alcian blue and Eosin B. Sections show a well-defined polarized epithelium, with folding beginning in the intestinal bulb and extending into the upper intestine (black arrow). **b** The goblet cells are stained dark blue (black arrowheads) and intestines (stained purple) are clearly visible, while mucous production can be seen in the luminal space, stained light blue. **bi** This magnified inset from **b** shows enterocytes with large supranuclear vesicles (white arrowheads). **c** 10× magnification of 5-μm sections through 7 dpf *shank3abΔC* ^+/−^ mutants. Mucous production can be seen in luminal space shown in 40× magnification of **d**, along with goblet cells (black arrowheads) and intestinal lumen. **di** Similar to what is seen in WT fish, *shank3abΔC*^+/−^ mutants display enterocytes with large supranuclear vesicles (white arrowheads). **e** Comparison of WT and *shank3abΔC* ^+/−^ mutant goblet cell count; no significant difference was found at 7 dpf

In order to assess the integrity of both WT and *shank3abΔC*^*+/−*^ mutant gut tissues, we processed 7 dpf larvae for paraffin sectioning. Larvae were sectioned along the sagittal plane at a thickness of 5 μm and stained with alcian blue and Eosin B as a counterstain, with a left-right anterior to posterior orientation (Fig. 6). Typical features of gut morphology at this stage of development are captured in WT sections (Fig. 6a, b). The intestine is comprised of polarized epithelium and is well-defined with dense cytoplasm. Folding of the epithelium (plicae) can be seen readily in the intestinal bulb extending into the upper-intestine (arrows), but these plicae do not extend any significant distance into the luminal space. Goblet cells and mucin production can be seen throughout the upper-intestine (black arrowheads). In *shank3abΔC* ^+/−^ larval sections (Fig. 6c, d), like that seen in WT, folding can be seen in the intestinal bulb, and extending into the upper-intestine; goblet cells and heavy mucin production can be seen throughout the upper-intestine (black arrowheads); and enterocytes have similar large supranuclear vesicles (white arrowheads) [65]. Statistically, *shank3abΔC* ^*+/−*^ larvae did not show a significant increase in goblet cells (*p* = 0.750, *n* = 25 for *shank3abΔC* ^*+/−*^ fish, *n* = 15 for WT).

We also quantified goblet cells in adult DTs from adult (> 1 year old) WT, *shank3abΔ*^*+/−*^, and *shank3abΔC*^*−/−*^ animals that had been sacrificed to acquire adequate CNS tissue for western analysis. Paraffin sections were collected near the IB and UI transition zone and stained similar to 7 dpf larvae. In contrast to larvae, goblet cell counts from adult gut tissue were significantly increased in *shank3abΔC* ^*+/−*^ and *shank3abΔC* ^*−/−*^ mutant as compared to WT (Additional file 1: Figure S2; *p* < 0.001, *n* = 6 for WT, *n* = 6 for *shank3abΔC* ^*+/−*^, and *n* = 6 for *shank3abΔC* ^*−/−*^).

### Larval shank3abΔC ^+/−^ and shank3abΔC ^−/−^ DTs contain fewer serotonin (5-HT+) cells

**Fig. 7:**
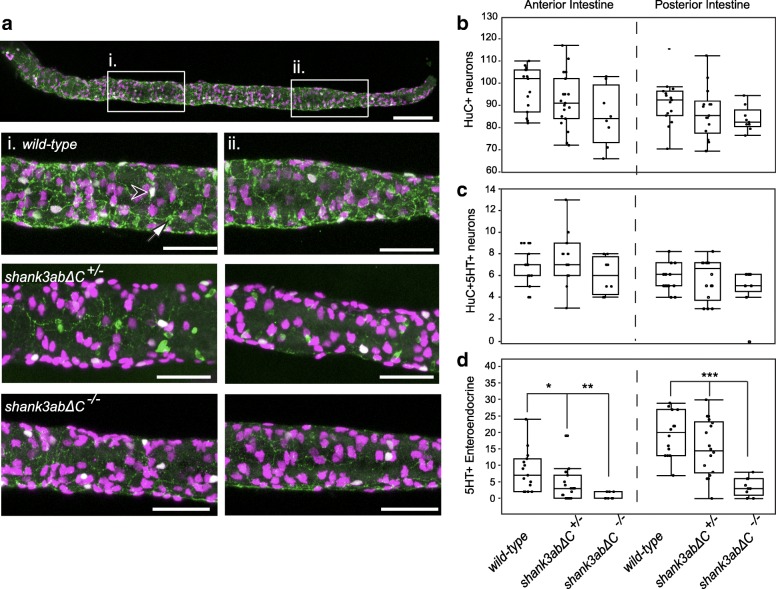
*Shank3abΔC* mutants have a reduction in enteroendocrine cells. **a** Anterior (i.) and posterior (ii.) regions were sampled to quantify the number of enteric neurons (HuC, magenta), serotonin enteric neurons (HuC+/5-HT+, white, black arrowhead), and enteroendocrine cells (5-HT+, green, white arrow). *Shank3abΔC* mutants did not show a significant decrease in either **b** enteric neurons or **c** serotonin positive enteric neurons. **d** Serotonin positive enteroendocrine cells were significantly decreased in *shank3abΔC* mutants for both anterior and posterior regions. WT, *n* = 15;*shank3abΔC* ^+/−^, *n* = 19; *shank3abΔC* ^−/−^, *n* = 9. Scale bars represent 100 μm (**a**) and 50 μm (i, ii)

To determine whether impaired gut motility was due to deficits in the ENS or enteroendocrine cells (EECs), *shank3abΔC* mutants and WT larvae were immunostained for the pan-neuronal marker HuC and 5-HT (Fig. 7, WT, *n* = 15; *shank3abΔC* ^+/−^, *n* = 19; *shank3abΔC* ^−/−^, *n* = 9). Enteric neurons (HuC+), serotonin enteric neurons (HuC+/5-HT+), and enteroendocrine (5-HT+) cell bodies from representative anterior and posterior regions (Fig. 7a, (i and ii)) were counted using ImageJ and confocal Z-stacks. There was no significant decrease in enteric neurons along the intestinal tract of *shank3abΔC* mutants compared to WT larvae. However, there was a significant decrease in serotonin-positive EECs in the intestinal tract of *shank3abΔC*^*+/−*^ (anterior, *p* = 0.0128; posterior, *p* = 0.0003) and *shank3abΔC*^*−/−*^ (anterior, *p* = 0.0013; posterior *p* < 0.0001) larvae compared to WT. EECs are mechanosensitive cells that release serotonin in response to mechanical and/or chemical stimulation, which in turn causes the intestine to increase mucus secretion and peristalsis, both of which facilitate transit [66].

## Discussion

### Zebrafish as a model system for GI distress in ASD

Our studies are the first to establish DT dysmotility as a robust phenotype in any *SHANK3* mutant animal model of ASD. The developmental accessibility and transparency of the zebrafish model system allows highly quantitative assays to be carried out in an intact animal [40, 46]. This type of systems-level analysis is important because GI motility is coordinately regulated by diverse neuronal tissues [67]. For example, the central nervous system (CNS) and parasympathetic ganglia increase GI motility in anticipation of the arrival of food; the vagus nerve releases acetylcholine to promote stomach emptying [53–56]; and coordinated regulation by the enteric nervous system [36, 68–70], enterochromaffin cells [71, 72], interstitial cells of Cajal [73], and microbiome [74–76] all help to promote motility and digestion. In the zebrafish larva, it is possible to quantify this coordinated regulation as food passes through the DT tract. Our analyses of DT function in WT and *shank3abΔC* zebrafish reveal hypomotility as a phenotype with likely parallels to GI distress described in individuals with PMS. Based on our findings, our working hypothesis is that mutations in *shank3ab* disrupt EEC regulation of DT motility.

### Zebrafish Shank3ab proteins show similar isoform complexity and post-synaptic density enrichment to that seen in mammals

Zebrafish and mouse both express multiple Shank3 isoforms in the brain. Previous studies of the mouse *Shank3* gene have identified ten to twelve *Shank3* isoforms arising from five transcriptional start sites as well as alternative splicing [62, 77]. While zebrafish *shank3ab* genes have also been shown to encode at least six isoforms [21], these likely represent an underestimate based on our western data. Despite using an antibody targeting the C-terminus of Shank3 that would only recognize three of the six previously characterized splice-variants, our western identifies five abundant and additional, less abundant Shank3 isoforms that range in size from 75 to 250 kD. These bands are absent in brain-specific PSD extracts from *shank3abΔC* ^*−/−*^ animals, showing that these bands represent specific Shank3ab protein products. This antibody recognizes residues downstream of the induced mutations; therefore, it is possible that there is residual Shank3 protein in our zebrafish *shank3ΔC* ^*−/−*^ mutants. Future experiments are needed to assess the full complement of protein products produced by *shank3ab* genes as they relate to different phenotypes associated with the *shank3abΔC* mutant genotype.

Immunohistochemistry of larval cerebellar tissue shows that zebrafish Shank3ab proteins co-localize with PSD-95 at excitatory post-synaptic densities, as has been reported previously in mammalian cultured hippocampal neurons [78]. The lack of Shank3ab puncta in *shank3abΔC* mutants shows that the mammalian Shank3 antibody also specifically recognizes zebrafish Shank3ab in tissue. Taken together, these findings support that there is functional conservation between zebrafish and mammalian Shank3 proteins.

### Mutations in *shank3ab* result in DT hypomotility

Functional comparisons of WT and *shank3abΔC* ^*+/−*^ mutant larvae show significantly reduced DT peristaltic frequencies with correspondingly prolonged DT transit times. It is important to note that our assay did not test appetite or potential differences in jaw shape that might impact their ability to forage. Comparisons of overall size between WT and *shank3abΔC* mutant larvae found no significant differences (data not shown), but further study of aspects such as jaw size/shape and potential differences in appetite should be investigated in future research. Although we saw no signs of regurgitation in larval *shank3abΔC* mutants, fluorescent microspheres remained in the intestinal bulb for prolonged periods of time, and in a small number of cases, fluorescent microspheres moved back into the pharynx in *shank3abΔC* ^*+/−*^ individuals, a behavior not seen in the WT larvae. Such prolonged transit times and longer dwell times in anterior DT in *shank3abΔC* ^*+/−*^ larvae are consistent with reflux and vomiting in individuals with PMS [11, 12] and phenotypes reported in a zebrafish *chd7* mutant model of CHARGE syndrome [79].

### Hypomotility in *shank3abΔC*^+/−^ larvae can be partially rescued by human SHANK3 mRNA

Prolonged dwell times in the intestinal bulb of 7-day-old *shank3abΔC* ^*+/−*^ larvae could be partially rescued by injecting human SHANK3 mRNA at the one cell stage. Human *SHANK3* RNA specifically rescued transit from the intestinal bulb (analogous to the mammalian stomach) to the upper intestine but not overall intestinal motility. Unlike endogenous transcripts, RNAs injected at fertilization are expressed transiently, for approximately 48 h, throughout the embryo [80]. Therefore, such a manipulation would likely rescue early *shank3ab*-dependent processes like hindbrain morphogenesis [20], but perhaps not later *shank3ab*-dependent processes like ENS migration, EEC differentiation, growth and function, and general epithelial maintenance.

### Structurally intact DT tissue in *shank3ab* mutant larvae contains fewer serotonin-expressing enteroendocrine cells

Histological analysis of *shank3abΔC* ^*+/−*^ and WT larval DTs reveals normal tissue structure in mutants. Despite the fact that inflammation and leaky gut have been implicated in ASD-linked GI distress [81, 82], at least at larval stages, *shank3abΔC* ^+/−^ zebrafish exhibited an intact columnar epithelium and similar numbers of goblet cells to their WT counterparts and to the literature. Likewise, analysis of the larval ENS showed similar numbers of enteric neurons in WT and *shank3abΔC* mutants.

By staining for serotonin in the DT, we showed that EECs are significantly reduced in both *shank3abΔC* ^+/−^ and *shank3abΔC* ^−/−^ larvae. EECs in the gut synthesize 95% of the body’s serotonin and, during digestion, serotonin release helps to coordinate secretion and motility as well as satiety and pain sensation [83, 84]. RNA-seq analysis from cells dissociated and sorted from 6 dpf WT larvae show *shank3a* and *shank3b* are detectable in EECs as well other intestinal epithelial cells, with *shank3a* expressed at higher levels than *shank3b* (personal communication from Lihua Ye, Roger A. Liddle, and John F. Rawls, Duke University). Therefore, Shank3 dosage-dependent reductions in DT EECs could help to explain dysmotility phenotypes. Future experiments are required to establish a causal relationship between EEC reductions and DT dysmotility phenotypes. Additionally, future studies will need to explore other cell types, such as interstitial cells of Cajal and smooth muscle cells, as defects in these may also explain the observed motility phenotype.

Unlike larval *shank3abΔC* ^*+/−*^, DTs from both *shank3abΔC* ^*+/−*^ and *shank3abΔC* ^*−/−*^ adults show significantly more goblet cells. Such a goblet cell expansion could result from disrupting Delta-Notch signaling and/or increasing inflammation. As in mammals, zebrafish goblet cell differentiation (and DT epithelial maintenance in general) is reliant on Delta-Notch signaling [85, 86]; therefore, it is possible that Shank3ab normally interacts with Delta-Notch signaling in a way that is pivotal to GI differentiation and maintenance. Recent research reports such interactions between *ascl1a* (a transcription factor important for neuronal commitment and differentiation) and Delta-Notch have drastic impacts on both motility and secretory cell expression [87]. An alternate possibility is that reduced motility feeds back on microbial communities to cause inflammation [88]. Zebrafish serve as an excellent model system for studying inflammation, and it has been observed that such inflammation can cause a dramatic expansion of goblet cells [89]. Importantly, inflammation is reported to occur in ASD, and the potential impact it might have on patients is not yet known [90]. Distinguishing between these possibilities and expanding on their potential impact is an area of interest for future studies.

## Conclusions

The *shank3abΔC* mutant zebrafish generated herein mimics clinically relevant mutations shown previously to cause Phelan-McDermid syndrome. Westerns and immunohistochemistry show zebrafish Shank3 to be functionally conserved with mammalian Shank3 in terms of isoform complexity and enrichment at post-synaptic sites in the cerebellum, validating the zebrafish model system. *shank3abΔC* mutant zebrafish show reduced DT peristalsis, increased digestive transit time, and reductions in EECs consistent with reduced GI functionality and symptoms of GI distress reported in people with Phelan-McDermid syndrome. Prolonged passage time in *shank3abΔC* mutants can be rescued by human *SHANK3* mRNA establishing reduced Shank3 in the zebrafish model system as causal for GI dysmotility. This zebrafish model system provides a basis for studying the functional relationships between the brain, gut, and microbiome as these relate to GI distress common in Phelan-McDermid syndrome.

## Additional files

Additional file 1: **Figure S1.** Insertion mutations in zebrafish *shank3* orthologues produce unique restriction maps for genotyping. **Figure S2.** a Transverse 5 μm section of adult WT upper intestinal tissue stained with alcian blue and Eosin B (*n* = 6 for WT, *n* = 6 for *shank3abΔC* ^*+/−*^ and *n* = 6 for *shank3abΔC* ^*−/−*^). b 40x magnification shows dense plicae that extend to the point of nearly occluding the luminal space. c, d In *shank3abΔC* ^+/−^ upper intestinal tissue, increased counts of goblet cells (black arrowheads) suggest inflammation. e, f Homozygous *shank3abΔC* ^−/−^ adults also show increased goblet cell count. **Table S1.** Oligonucleotides used for sgRNA synthesis. **Table S2.** Primers used for PCR and sanger sequencing. **Table S3.** % bead occupancy in WT and *shank3abΔC+/−* larvae at 3 h post-feed. **Table S4.** % bead occupancy in WT and *shank3abΔC+/−* larvae at 6 h post-feed. **Table S5.** % bead occupancy in WT and *shank3abΔC+/−* larvae at 12 h post-feed. **Table S6.** % bead occupancy in WT and *shank3abΔC+/−* larvae at 24 h post-feed. **Table S7.** Upper and lower GI rate of peristaltic period (seconds between contractions). **Table S8.** R script for Permutation. **Table S9.** *shank3abΔC+/−* injected with either Human *SHANK3* mRNA short (32T) or long (5 T) isoform, 3–24 h post feed. (DOCX 4255 kb)

## Abbreviations

5-HT: Serotonin
ASD: Autism spectrum disorder
CNS: Central nervous system
DT: Digestive tract
ENS: Enteric nervous system
GI: Gastrointestinal
PMS: Phelan-McDermid syndrome
WT: Wild type
