# Supplementary material for: Intestinal dysmotility in a zebrafish (Danio rerio) shank3a;shank3b mutant model of autism
Source: Mol Autism. 2019 Jan 31;10:3. doi: 10.1186/s13229-018-0250-4 (PMC6357389; doi:10.1186/s13229-018-0250-4)
Supplement: Supplementary file 3 — Figure S1. Insertion mutations in zebrafish shank3 orthologues produce unique restriction maps for genotyping. Figure S2. a Transverse 5 μm section of adult WT upper intestinal tissue stained with alcian blue and Eosin B (n = 6 for WT, n = 6 for shank3abΔC +/− and n = 6 for shank3abΔC −/−). b 40x magnification shows dense plicae that extend to the point of nearly occluding the luminal space. c, d In shank3abΔC +/− upper intestinal tissue, increased counts of goblet cells (black arrowheads) suggest inflammation. e, f Homozygous shank3abΔC −/− adults also show increased goblet cell count. Table S1. Oligonucleotides used for sgRNA synthesis. Table S2. Primers used for PCR and sanger sequencing. Table S3. % bead occupancy in WT and shank3abΔC+/− larvae at 3 h post-feed. Table S4. % bead occupancy in WT and shank3abΔC+/− larvae at 6 h post-feed. Table S5. % bead occupancy in WT and shank3abΔC+/− larvae at 12 h post-feed. Table S6. % bead occupancy in WT and shank3abΔC+/− larvae at 24 h post-feed. Table S7. Upper and lower GI rate of peristaltic period (seconds between contractions). Table S8. R script for Permutation. Table S9. shank3abΔC+/− injected with either Human SHANK3 mRNA short (32T) or long (5 T) isoform, 3–24 h post feed. (DOCX 4255 kb) [file 13229_2018_250_MOESM1_ESM.docx]

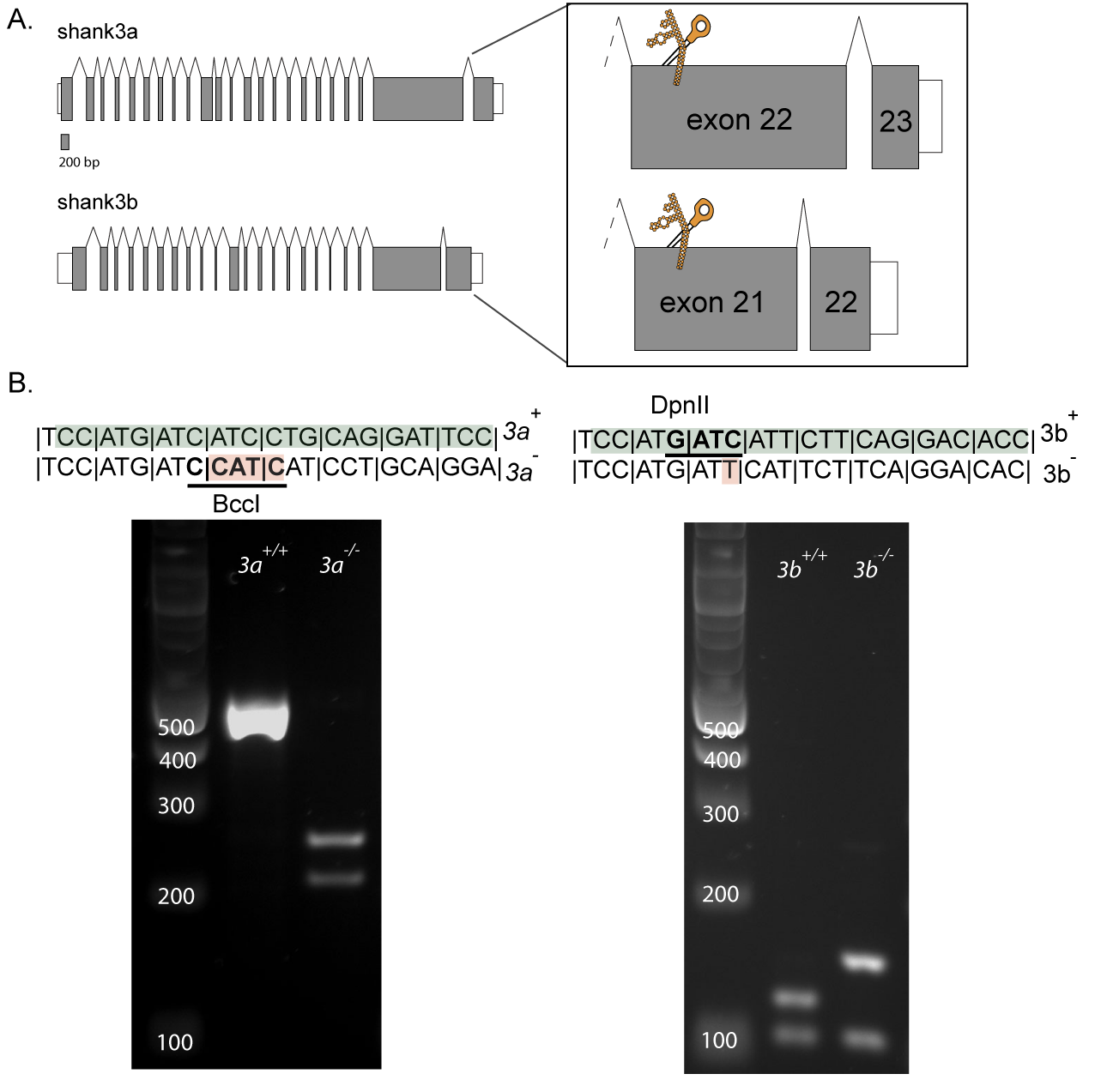


**Figure S1. Insertion mutations in zebrafish *shank3* orthologues produce unique restriction maps for genotyping.** A. Exon-Intron diagram showing the second to last exon in *shank3a* and *shank3b* targeted by CRISPR-Cas9; boxes represent exons and lines represent introns (note introns not drawn to scale). Inset shows portion of exon with scissors representing the targeted Cas9 cut site. Guide sequences are highlighted in green and mutations are highlighted in red. Restriction sites for detecting mutations are underlined. B. Sequences of target sites (green) and restriction sites (bold) used to produce digestion patterns for genotyping. Restriction digests show the mutational insertion of a restriction site for *shank3a* and the destruction of a restriction site for *shank3b*.


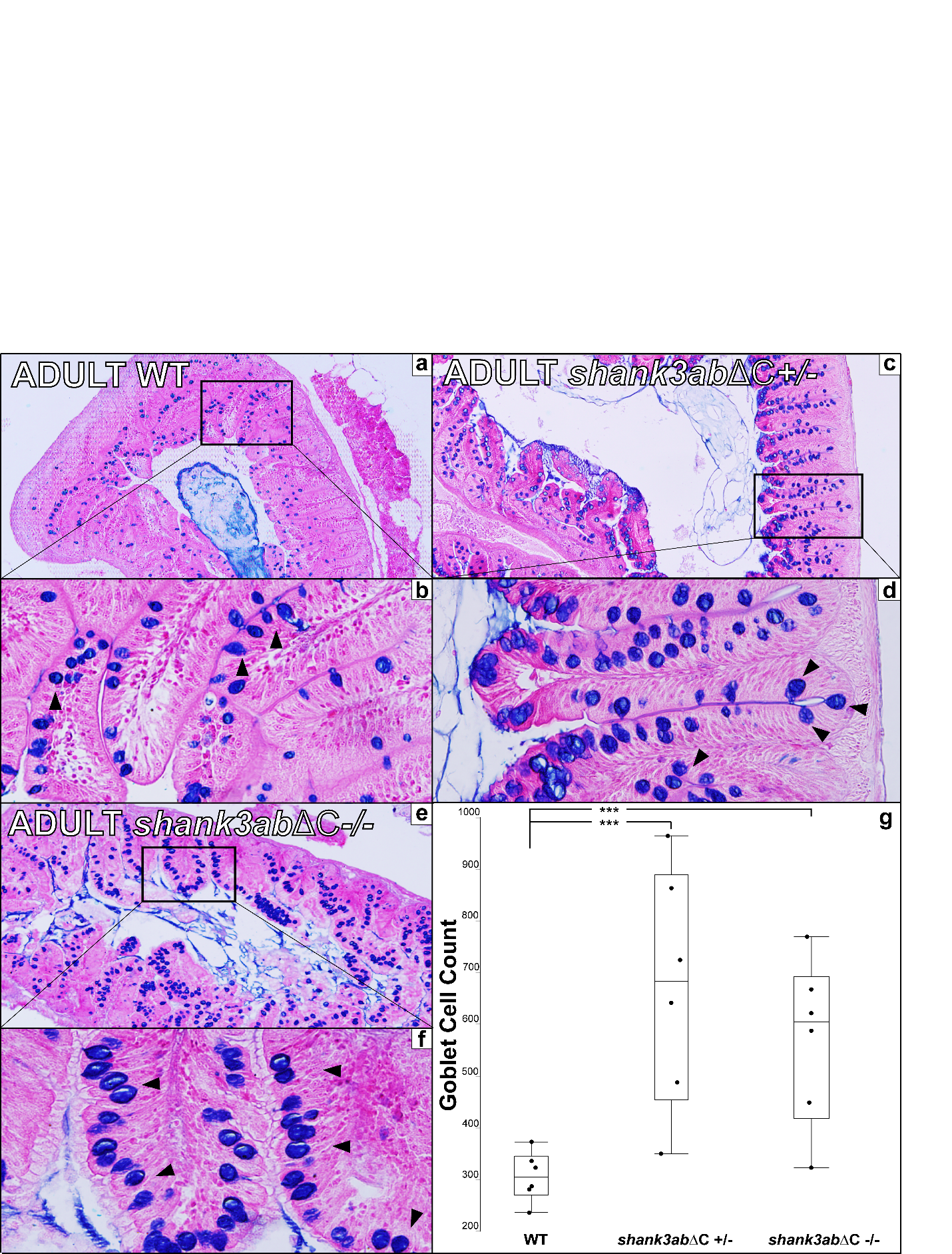


**Figure S2 a** Transverse 5μm section of adult WT upper intestinal tissue stained with alcian blue and Eosin B (n=6 for WT, n=6 for *shank3abΔC ^+/-^* and n=6 for *shank3abΔC ^-/-^)*. **b** 40x magnification shows dense plicae that extend to the point of nearly occluding the luminal space. **c,d** In *shank3abΔC* ^+/-^ upper intestinal tissue, increased counts of goblet cells (black arrowheads) suggest inflammation. **e,f** Homozygous *shank3abΔC* ^-/-^ adults also show increased goblet cell count. Comparison of goblet cell counts in WT, *shank3abΔC* ^+/-^, and *shank3abΔC* ^-/-^ adults show a significant increase in the number of goblet cells in both *shank3abΔC* ^+/-^ and *shank3abΔC* ^-/-^ adults. WT and *shank3abΔC ^-/-^* comparison (p<0.0138) and WT and *shank3abΔC ^+/-^* comparison (p<0.0015).

**Adult goblet cell count is increased in *shank3* mutants.**

**Table S1. Oligonucleotides used for sgRNA synthesis.**

| Name | Sequence |
| --- | --- |
| *shank3a* exon 22 | TAATACGACTCACTATAGGGAATCCTGCAGGATGATCAGTTTTAGAGCTAGAAATAGC |
| *shank3b* exon 21 | TAATACGACTCACTATAGGGTGTCCTGAAGAATGATCAGTTTTAGAGCTAGAAATAGC |
| Universal tracRNA backbone | AAAAGCACCGACTCGGTGCCACTTTTTCAAGTTGATAACGGACTAGCCTTATTTTAACTTGCTATTTCTAGCTCTAAAAC |

**Table S2. Primers used for PCR and sanger sequencing.**

| Primer | Sequence |
| --- | --- |
| *shank3a* exon 22 forward | GAGACTACATGCATTGCCTCAG |
| *shank3a* exon 22 reverse | GAGTTGGCGTGAACATACCC |
| *shank3b* exon 21 forward | CACCTCCATCCTTTCTTCCA |
| *shank3b* exon 21 reverse | GCATACGGATTCTCCACAGG |

| *shank3abΔC+/-* and WT bead transit at 3hr post feed | | | | | | | | | | |
| --- | --- | --- | --- | --- | --- | --- | --- | --- | --- | --- |
| n | Pharynx WT | Pharynx *shank3* | Int bulb WT | Int bulb *shank3* | Upper int WT | Upper int *shank3* | Lower int WT | Lower int *shank3* | Expelled WT | Expelled *shank3* |
| 1 | 0 | 0 | 5.1 | 76.2 | 61.6 | 23.8 | 33.3 | 0 | 0 | 0 |
| 2 | 0 | 0 | 46 | 0 | 2.3 | 100 | 38.8 | 0 | 12.9 | 0 |
| 3 | 0 | 0 | 100 | 4.6 | 0 | 95.4 | 0 | 0 | 0 | 0 |
| 4 | 0 | 0 | 0 | 71.5 | 0 | 28.5 | 100 | 0 | 0 | 0 |
| 5 | 0 | 0 | 21.9 | 100 | 78.1 | 0 | 0 | 0 | 0 | 0 |
| 6 | 0 | 0 | 65.4 | 64.1 | 34.6 | 35.9 | 0 | 0 | 0 | 0 |
| 7 | 0 | 0 | 78.4 | 16.1 | 17.6 | 83.9 | 4 | 0 | 0 | 0 |
| 8 | 0 | 0 | 30.2 | 0 | 15 | 100 | 54.8 | 0 | 0 | 0 |
| 9 | 0 | 0 | 100 | 82 | 0 | 18 | 0 | 0 | 0 | 0 |
| 10 | 0 | 0 | 100 | 100 | 0 | 0 | 0 | 0 | 0 | 0 |
| 11 | 0 | 17 | 95.5 | 83 | 0 | 0 | 4.5 | 0 | 0 | 0 |
| 12 | 0 | 0 | 92.5 | 93 | 0 | 5 | 7.5 | 2 | 0 | 0 |
| 13 | 0 | 7 | 47.2 | 30 | 52.8 | 63 | 0 | 0 | 0 | 0 |
| 14 |  | 1 |  | 77 |  | 22 |  | 0 |  | 0 |
| 15 |  | 0 |  | 93 |  | 7 |  | 0 |  | 0 |
|  | Pharynx WT | Pharynx *shank3* | Int bulb WT | Int bulb *shank3* | Upper int WT | Upper int *shank3* | Lower int WT | Lower int *shank3* | Expelled WT | Expelled *shank3* |
| avg | 0 | 1.66 | 60.16 | 59.36 | 20.15 | 38.83 | 18.68 | 0.13 | 0.99 | 0 |

**Table S3. % bead occupancy in WT and *shank3abΔC+/-* larvae at 3 hours post-feed**

**Table S4. % bead occupancy in WT and *shank3abΔC+/-* larvae at 6 hours post-feed**

| *shank3abΔC+/-* and WT bead transit at 6hr post feed | | | | | | | | | | | |
| --- | --- | --- | --- | --- | --- | --- | --- | --- | --- | --- | --- |
| n | Pharynx WT | Pharynx *shank3* | Int bulb WT | Int bulb *shank3* | Upper int WT | Upper int *shank3* | Lower int WT | Lower int *shank3* | Expelled WT | Expelled *shank3* |  |
| 1 | 0 | 0 | 0 | 100 | 0 | 0 | 4.4 | 0 | 95.6 | 0 |  |
| 2 | 0 | 0 | 0 | 3.3 | 3.4 | 96.7 | 26.2 | 0 | 70.4 | 0 |  |
| 3 | 0 | 0 | 100 | 6.9 | 0 | 93.1 | 0 | 0 | 0 | 0 |  |
| 4 | 0 | 0 | 0 | 90.2 | 0 | 9.8 | 0 | 0 | 100 | 0 |  |
| 5 | 0 | 0 | 18.9 | 100 | 9.4 | 0 | 0 | 0 | 71.7 | 0 |  |
| 6 | 0 | 0 | 51.2 | 68 | 17.2 | 32 | 8.9 | 0 | 22.7 | 0 |  |
| 7 | 0 | 0 | 21.5 | 38.1 | 9.3 | 61.9 | 52.1 | 0 | 17.1 | 0 |  |
| 8 | 0 | 0 | 0 | 0 | 0 | 100 | 0 | 0 | 100 | 0 |  |
| 9 | 0 | 0 | 51 | 79 | 0 | 21 | 0 | 0 | 49 | 0 |  |
| 10 | 0 | 0 | 100 | 95 | 0 | 5 | 0 | 0 | 0 | 0 |  |
| 11 | 0 | 13 | 66 | 87 | 0 | 0 | 34 | 0 | 0 | 0 |  |
| 12 | 0 | 0 | 25.6 | 93 | 0 | 4 | 74.4 | 3 | 0 | 0 |  |
| 13 | 0 | 0 | 26.3 | 21 | 73.7 | 79 | 0 | 0 | 0 | 0 |  |
| 14 |  | 0 |  | 83 |  | 13 |  | 4 |  | 0 |  |
| 15 |  | 0 |  | 94 |  | 6 |  | 0 |  | 0 |  |
|  | Pharynx WT | Pharynx *shank3* | Int bulb WT | Int bulb *shank3* | Upper int WT | Upper int *shank3* | Lower int WT | Lower int *shank3* | Expelled WT | Expelled *shank3* |  |
| avg | 0 | 0.86 | 35.34 | 63.90 | 8.69 | 34.76 | 15.38 | 0.46 | 40.57 | 0 |  |

**Table S5. % bead occupancy in WT and *shank3abΔC+/-* larvae at 12 hours post-feed**

| *shank3abΔC+/-* and WT bead transit at 12hr post feed | | | | | | | | | | | |
| --- | --- | --- | --- | --- | --- | --- | --- | --- | --- | --- | --- |
| n | Pharynx WT | Pharynx *shank3* | Int bulb WT | Int bulb *shank3* | Upper int WT | Upper int *shank3* | Lower int WT | Lower int *shank3* | Expelled WT | Expelled *shank3* |  |
| 1 | 0 | 0 | 0 | 93.3 | 0 | 6.7 | 0 | 0 | 100 | 0 |  |
| 2 | 0 | 0 | 0 | 0 | 0 | 0 | 0 | 0 | 100 | 100 |  |
| 3 | 0 | 0 | 17 | 4.9 | 6.7 | 88.1 | 17.2 | 7 | 59.1 | 0 |  |
| 4 | 0 | 0 | 0 | 50.4 | 0 | 39 | 0 | 0 | 100 | 10.6 |  |
| 5 | 0 | 0 | 0 | 71.4 | 0 | 8.1 | 0 | 0 | 100 | 20.5 |  |
| 6 | 0 | 0 | 32.9 | 44.2 | 28.4 | 55.8 | 0 | 0 | 38.7 | 0 |  |
| 7 | 0 | 8 | 18.9 | 62.9 | 0 | 29.1 | 0 | 0 | 81.1 | 0 |  |
| 8 | 0 | 0 | 7 | 0 | 0 | 100 | 0 | 0 | 93 | 0 |  |
| 9 | 0 | 0 | 48.3 | 94 | 0 | 6 | 0 | 0 | 51.7 | 0 |  |
| 10 | 0 | 0 | 65.4 | 98 | 0 | 2 | 0 | 0 | 34.6 | 0 |  |
| 11 | 0 | 0 | 100 | 88 | 0 | 12 | 0 | 0 | 0 | 0 |  |
| 12 | 0 | 0 | 76.3 | 87 | 0 | 10 | 0 | 3 | 23.7 | 0 |  |
| 13 | 0 | 0 | 18.3 | 12 | 0 | 88 | 0 | 0 | 81.7 | 0 |  |
| 14 |  | 0 |  | 83 |  | 13 |  | 4 |  | 0 |  |
| 15 |  | 0 |  | 97 |  | 3 |  | 0 |  | 0 |  |
|  | Pharynx WT | Pharynx *shank3* | Int bulb WT | Int bulb *shank3* | Upper int WT | Upper int *shank3* | Lower int WT | Lower int *shank3* | Expelled WT | Expelled *shank3* |  |
| avg | 0 | 0.53 | 29.54 | 59.07 | 2.7 | 30.72 | 1.32 | 0.93 | 66.43 | 8.74 |  |

**Table S6. % bead occupancy in WT and *shank3abΔC+/-* larvae at 24 hours post-feed**

| *shank3abΔC+/-* and WT bead transit at 24hr post feed | | | | | | | | | | | |
| --- | --- | --- | --- | --- | --- | --- | --- | --- | --- | --- | --- |
| n | Pharynx WT | Pharynx *shank3* | Int bulb WT | Int bulb *shank3* | Upper int WT | Upper int *shank3* | Lower int WT | Lower int *shank3* | Expelled WT | Expelled *shank3* |  |
| 1 | 0 | 0 | 0 | 0 | 0 | 0 | 0 | 0 | 100 | 100 |  |
| 2 | 0 | 0 | 0 | 0 | 0 | 0 | 0 | 0 | 100 | 100 |  |
| 3 | 0 | 0 | 0 | 0 | 0 | 0 | 0 | 0 | 100 | 100 |  |
| 4 | 0 | 0 | 0 | 0 | 0 | 0 | 0 | 10 | 100 | 90 |  |
| 5 | 0 | 0 | 0 | 0 | 0 | 0 | 0 | 0 | 100 | 100 |  |
| 6 | 0 | 0 | 0 | 0 | 0 | 0 | 0 | 13.2 | 100 | 86.8 |  |
| 7 | 0 | 7 | 18.8 | 0 | 0 | 68.3 | 0 | 0 | 81.2 | 24.7 |  |
| 8 | 0 | 0 | 0 | 0 | 0 | 0 | 0 | 0 | 100 | 100 |  |
| 9 | 0 | 0 | 0 | 0 | 0 | 0 | 0 | 0 | 100 | 100 |  |
| 10 | 0 | 0 | 0 | 0 | 0 | 0 | 0 | 0 | 100 | 100 |  |
| 11 | 0 | 0 | 0 | 0 | 0 | 0 | 0 | 0 | 100 | 100 |  |
| 12 | 0 | 0 | 0 | 0 | 0 | 0 | 0 | 0 | 100 | 100 |  |
| 13 | 0 | 0 | 0 | 0 | 0 | 0 | 0 | 0 | 100 | 100 |  |
| 14 |  | 0 |  | 0 |  | 0 |  | 0 |  | 100 |  |
| 15 |  | 0 |  | 0 |  | 0 |  | 0 |  | 100 |  |
|  | Pharynx WT | Pharynx *shank3* | Int bulb WT | Int bulb *shank3* | Upper int WT | Upper int *shank3* | Lower int WT | Lower int *shank3* | Expelled WT | Expelled *shank3* |  |
| avg | 0 | 0.46 | 1.44 | 0 | 0 | 4.55 | 0 | 1.54 | 98.55 | 93.43 |  |

**Table S7. Upper and lower GI rate of peristaltic period (seconds between contractions)**

|  | upper GI tract | |  | lower GI tract | |
| --- | --- | --- | --- | --- | --- |
|  | *shank3abΔC+/-* | WT |  | *shank3abΔC+/-* | WT |
|  | 46.889 | 47.143 |  | 46.889 | 47.143 |
|  | 52.5 | 38.25 |  | 52.5 | 38.25 |
|  | 70 | 31 |  | 70 | 31 |
|  | 60.667 | 36.34 |  | 60.667 | 40 |
|  | 44.33 | 36.64 |  | 44.33 | 40 |
|  | 51.33 | 33 |  | 51.33 | 33 |
|  | 61.2 | 38.833 |  | 61.2 | 38.833 |
|  | 25.397 | 37.667 |  | 25.397 | 37.667 |
|  | 20.207 | 38.909 |  | 60.8 | 38.909 |
|  | 85 | 39.667 |  | 85 | 39.667 |
|  | 60.4 | 51.634 |  | 60.4 | 51.634 |
|  | 61.2 | 39.4 |  | 61.2 | 39.4 |
|  | 51.33 | 44.5 |  | 51.33 | 44.5 |
|  | 61.2 | 47.818 |  | 61.2 | 47.818 |
|  | 51.333 | 39.714 |  | 51.333 | 39.714 |
|  | 49.333 | 42.615 |  | 49.333 | 42.615 |
|  | 40.5 | 40.923 |  | 40.5 | 40.923 |
|  | 44.571 | 36.2 |  | 44.571 | 36.2 |
|  | 53.2 | 35.067 |  | 53.2 | 35.067 |
|  |  | 38 |  |  | 38 |
| avg: | 52.1361579 | 39.666 |  | 54.272632 | 40.017 |
| p value: | p < 0.0009 | |  | p < 0.0001 | |

**Table S8. R script for Permutation**

| # Standard Anova on these data, "a" is the dataset  mod1 <- lm(a$percentage ~ a$Region + a$Time + a$Gen + a$Region:a$Gen + a$Region:a$Time + a$Gen:a$Time + a$Gen:a$Region:a$Time + (1+a$Time\|a$ID))  #mod1 <- lmer(a$percentage ~ a$Region + a$Time + a$Gen + a$Region:a$Gen + a$Region:a$Time + a$Gen:a$Time + Error(a$ID/(a$Time)))  ANOVA <- summary(aov(mod1))  unlist_anova<-unlist(ANOVA)  unlist_anova<-as.numeric(unlist_anova)  cat( " The standard ANOVA for these data follows ","\n")  FRegion <- unlist_anova[25] # Saving F values for future use  FTime <- unlist_anova[26]  FGen <- unlist_anova[27]  #The following obtains the F values for the interactions  Fi1 <- unlist_anova[28]  Fi2 <- unlist_anova[29]  Fi3 <- unlist_anova[30]  Fi4<-unlist_anova[31]  print(ANOVA)  print( "Resampling as in Manly with unrestricted sampling of observations. ")  # Now start resampling  nreps <- 5000  Freg <- numeric(nreps) #Set up space to store F values as calculated.  Ftime <- numeric(nreps)  Fgen <- numeric(nreps)  fint1 <- numeric(nreps)  fint2 <- numeric(nreps)  fint3 <- numeric(nreps)  fint4 <- numeric(nreps)  Freg[1] <- FRegion  Ftime[1] <- FTime  Fgen[1] <- FGen  fint1[1] <- Fi1  fint2[1] <- Fi2  fint3[1] <- Fi3  fint4[1]<- Fi4  for (i in 2:nreps) {    lst <- split(a, a$ID)  a2 <- do.call(rbind, lst[sample(names(lst))])  row.names(a2) <- NULL  a2$Gen<-a$Gen    a3<-a2$percentage    mod2 <- lm(a3 ~ a2$Region + a2$Time + a2$Gen + a2$Region:a$Gen + a2$Region:a$Time + a2$Gen:a$Time + a2$Gen:a2$Region:a2$Time + (1+a2$Time\|a2$ID))  b <- summary(aov(mod2))  unlist_b<-unlist(b)  unlist_b<-as.numeric(unlist_b)  Freg[i] <- unlist_b[25]  Ftime[i] <- unlist_b[26]  Fgen[i] <- unlist_b[27]  fint1[i] <-unlist_b[28]  fint2[i] <- unlist_b[29]  fint3[i] <- unlist_b[30]  fint4[i] <- unlist_b[31]  }  probreg <- length(Freg[Freg >= FRegion + .Machine$double.eps ^0.5])/nreps  probtime <- length(Ftime[Ftime >= FTime+ .Machine$double.eps ^0.5])/nreps  probgen<-length(Fgen[Fgen >= FGen + .Machine$double.eps ^0.5])/nreps  probint1 <- length(fint1[fint1 >= Fi1 + .Machine$double.eps ^0.5])/nreps  probint2<-length(fint2[fint2 >= Fi2 + .Machine$double.eps ^0.5])/nreps  probint3<-length(fint3[fint3 >= Fi3 + .Machine$double.eps ^0.5])/nreps  probint4<-length(fint4[fint4 >= Fi4 + .Machine$double.eps ^0.5])/nreps  ### The addition of "+ .Machine$double.eps" is an aid against two numbers that differ only by  ### floating point computer calculations at the extreme.  cat(" The probability value for the interaction 1 is ",probint1, "\n")  cat(" The probability value for the interaction 2 is ",probint2, "\n")  cat(" The probability value for the interaction 3 is ",probint3, "\n")  cat(" The probability value for the interaction 4 is ",probint4, "\n")  cat(" The probability value for region is ", probreg, "\n")  cat(" The probability value for time is ", probtime, "\n")  cat(" The probability value for genotype is ", probgen, "\n") |
| --- |

**Table S9. *shank3abΔC+/-* injected with either Human SHANK3 mRNA short (32T) or long (5T) isoform, 3-24 hours post feed**

**3 hours post-feed:**

| **% bead occupancy in *shank3abΔC+/-* injected with either SHANK3 mRNA**  **short isoform (32t) or long isoform (5t)** | | | | | | | | | | |
| --- | --- | --- | --- | --- | --- | --- | --- | --- | --- | --- |
| n | pharynx  32T | pharynx  5T | int bulb 32T | int bulb 5T | upper int 32T | upper int 5T | lower int 32T | lower int 5T | Expelled 32T | Expelled 5T |
| 1 | 0 | 0 | 0 | 61 | 100 | 14 | 0 | 25 | 0 | 0 |
| 2 | 0 | 0 | 27 | 100 | 67 | 0 | 6 | 0 | 0 | 0 |
| 3 | 0 | 0 | 89 | 73 | 7 | 14 | 4 | 13 | 0 | 0 |
| 4 | 0 | 0 | 85 | 57 | 12 | 43 | 2 | 0 | 1 | 0 |
| 5 | 0 | 0 | 0 | 20 | 38 | 80 | 62 | 0 | 0 | 0 |
| 6 | 0 | 0 | 100 | 38 | 0 | 27 | 0 | 35 | 0 | 0 |
| 7 |  | 0 |  | 100 |  | 0 |  | 0 |  | 0 |
| 8 |  | 0 |  | 93 |  | 7 |  | 0 |  | 0 |
| 9 |  | 0 |  | 100 |  | 0 |  | 0 |  | 0 |
| 10 |  | 0 |  | 100 |  | 0 |  | 0 |  | 0 |
| 11 |  | 0 |  | 87 |  | 13 |  | 0 |  | 0 |
| 12 |  | 0 |  | 100 |  | 0 |  | 0 |  | 0 |
| 13 |  | 0 |  | 88 |  | 12 |  | 0 |  | 0 |
| 14 |  | 0 |  | 10 |  | 68 |  | 22 |  | 0 |
| 15 |  | 0 |  | 91 |  | 9 |  | 0 |  | 0 |
| 16 |  | 0 |  | 88 |  | 12 |  | 0 |  | 0 |
| 17 |  | 0 |  | 0 |  | 55 |  | 15 |  | 30 |
| 18 |  | 0 |  | 0 |  | 71 |  | 0 |  | 29 |
| 19 |  | 0 |  | 72 |  | 9 |  | 19 |  | 0 |
|  | pharynx  32T | pharynx  5T | int bulb 32T | int bulb 5T | upper int 32T | upper int 5T | lower int 32T | lower int 5T | Expelled 32T | Expelled 5T |
| average | 0 | 0 | 50 | 67 | 37 | 22 | 12 | 6 | 0 | 3 |

**6 hours post-feed:**

| **% bead occupancy in *shank3abΔC+/-* injected with either SHANK3 mRNA**  **short isoform (32t) or long isoform (5t)** | | | | | | | | | | | |
| --- | --- | --- | --- | --- | --- | --- | --- | --- | --- | --- | --- |
| n | pharynx  32t | pharynx  5t | int bulb 32t | int bulb 5t | upper int 32t | upper int 5t | lower int 32t | lower int 5t | Expelled 32t | Expelled 5t |  |
| 1 | 0 | 0 | 0 | 52 | 100 | 38 | 0 | 10 | 0 | 0 |  |
| 2 | 0 | 0 | 19 | 51 | 54 | 49 | 27 | 0 | 0 | 0 |  |
| 3 | 0 | 0 | 82 | 62 | 12 | 9 | 2 | 11 | 4 | 18 |  |
| 4 | 0 | 0 | 75 | 44 | 10 | 47 | 10 | 9 | 5 | 0 |  |
| 5 | 0 | 0 | 25 | 27 | 2 | 73 | 31 | 0 | 42 | 0 |  |
| 6 | 0 | 0 | 100 | 10 | 0 | 8 | 0 | 82 | 0 | 0 |  |
| 7 |  | 0 |  | 51 |  | 49 |  | 0 |  | 0 |  |
| 8 |  | 0 |  | 82 |  | 0 |  | 18 |  | 0 |  |
| 9 |  | 0 |  | 100 |  | 0 |  | 0 |  | 0 |  |
| 10 |  | 0 |  | 51 |  | 49 |  | 0 |  | 0 |  |
| 11 |  | 0 |  | 48 |  | 52 |  | 0 |  | 0 |  |
| 12 |  | 0 |  | 0 |  | 100 |  | 0 |  | 0 |  |
| 13 |  | 0 |  | 93 |  | 7 |  | 0 |  | 0 |  |
| 14 |  | 0 |  | 0 |  | 80 |  | 20 |  | 0 |  |
| 15 |  | 0 |  | 75 |  | 25 |  | 0 |  | 0 |  |
| 16 |  | 0 |  | 59 |  | 41 |  | 0 |  | 0 |  |
| 17 |  | 0 |  | 8 |  | 39 |  | 3 |  | 50 |  |
| 18 |  | 0 |  | 0 |  | 0 |  | 0 |  | 100 |  |
| 19 |  | 0 |  | 59 |  | 36 |  | 5 |  | 0 |  |
|  | pharynx  32t | pharynx  5t | int bulb 32t | int bulb 5t | upper int 32t | upper int 5t | lower int 32t | lower int 5t | Expelled 32t | Expelled 5t |  |
| average | 0 | 0 | 50 | 45 | 30 | 36 | 12 | 8 | 9 | 8 |  |

**12 hours post-feed:**

| **% bead occupancy in *shank3abΔC+/-* injected with either SHANK3 mRNA**  **short isoform (32t) or long isoform (5t)** | | | | | | | | | | | |
| --- | --- | --- | --- | --- | --- | --- | --- | --- | --- | --- | --- |
| n | pharynx  32t | pharynx  5t | int bulb 32t | int bulb 5t | upper int 32t | upper int 5t | lower int 32t | lower int 5t | Expelled 32t | Expelled 5t |  |
| 1 | 0 | 0 | 0 | 52 | 100 | 8 | 0 | 3 | 0 | 37 |  |
| 2 | 0 | 0 | 0 | 100 | 73 | 0 | 27 | 0 | 0 | 0 |  |
| 3 | 0 | 0 | 8 | 32 | 85 | 50 | 7 | 8 | 0 | 10 |  |
| 4 | 0 | 0 | 77 | 38 | 17 | 53 | 0 | 9 | 6 | 0 |  |
| 5 | 0 | 0 | 5 | 26 | 10 | 74 | 0 | 0 | 85 | 0 |  |
| 6 | 0 | 0 | 91 | 23 | 0 | 0 | 0 | 0 | 9 | 77 |  |
| 7 |  | 0 |  | 0 |  | 100 |  | 0 |  | 0 |  |
| 8 |  | 0 |  | 0 |  | 20 |  | 80 |  | 0 |  |
| 9 |  | 0 |  | 0 |  | 100 |  | 0 |  | 0 |  |
| 10 |  | 0 |  | 0 |  | 100 |  | 0 |  | 0 |  |
| 11 |  | 0 |  | 17 |  | 83 |  | 0 |  | 0 |  |
| 12 |  | 0 |  | 0 |  | 100 |  | 0 |  | 0 |  |
| 13 |  | 0 |  | 93 |  | 7 |  | 0 |  | 0 |  |
| 14 |  | 0 |  | 0 |  | 71 |  | 29 |  | 0 |  |
| 15 |  | 0 |  | 87 |  | 0 |  | 0 |  | 13 |  |
| 16 |  | 0 |  | 0 |  | 100 |  | 0 |  | 0 |  |
| 17 |  | 0 |  | 0 |  | 53 |  | 4 |  | 43 |  |
| 18 |  | 0 |  | 0 |  | 0 |  | 0 |  | 100 |  |
| 19 |  | 0 |  | 38 |  | 59 |  | 0 |  | 3 |  |
|  | pharynx  32t | pharynx  5t | int bulb 32t | int bulb 5t | upper int 32t | upper int 5t | lower int 32t | lower int 5t | Expelled 32t | Expelled 5t |  |
| average | 0 | 0 | 30 | 26 | 48 | 51 | 6 | 7 | 17 | 14 |  |

**24 hours post-feed:**

| **% bead occupancy in *shank3abΔC+/-* injected with either SHANK3 mRNA**  **short isoform (32t) or long isoform (5t)** | | | | | | | | | | | |
| --- | --- | --- | --- | --- | --- | --- | --- | --- | --- | --- | --- |
| n | pharynx  32t | pharynx  5t | int bulb 32t | int bulb 5t | upper int 32t | upper int 5t | lower int 32t | lower int 5t | Expelled 32t | Expelled 5t |  |
| 1 | 0 | 0 | 9 | 2 | 0 | 0 | 0 | 1 | 91 | 97 |  |
| 2 | 0 | 0 | 0 | 0 | 20 | 0 | 63 | 0 | 17 | 100 |  |
| 3 | 0 | 0 | 72 | 35 | 18 | 21 | 0 | 12 | 10 | 32 |  |
| 4 | 0 | 0 | 75 | 0 | 10 | 0 | 10 | 0 | 5 | 100 |  |
| 5 | 0 | 0 | 0 | 0 | 13 | 0 | 0 | 0 | 87 | 100 |  |
| 6 | 0 | 0 | 90 | 38 | 0 | 5 | 0 | 0 | 10 | 57 |  |
| 7 |  | 0 |  | 0 |  | 0 |  | 0 |  | 100 |  |
| 8 |  | 0 |  | 0 |  | 0 |  | 0 |  | 100 |  |
| 9 |  | 0 |  | 0 |  | 0 |  | 0 |  | 100 |  |
| 10 |  | 0 |  | 49 |  | 51 |  | 0 |  | 0 |  |
| 11 |  | 0 |  | 0 |  | 0 |  | 0 |  | 100 |  |
| 12 |  | 0 |  | 0 |  | 0 |  | 0 |  | 100 |  |
| 13 |  | 0 |  | 100 |  | 0 |  | 0 |  | 0 |  |
| 14 |  | 0 |  | 0 |  | 48 |  | 0 |  | 52 |  |
| 15 |  | 0 |  | 0 |  | 100 |  | 0 |  | 0 |  |
| 16 |  | 0 |  | 0 |  | 100 |  | 0 |  | 0 |  |
| 17 |  | 0 |  | 6 |  | 40 |  | 0 |  | 54 |  |
| 18 |  | 0 |  | 0 |  | 0 |  | 0 |  | 100 |  |
| 19 |  | 0 |  | 0 |  | 0 |  | 0 |  | 100 |  |
|  | pharynx  32t | pharynx  5t | int bulb 32t | int bulb 5t | upper int 32t | upper int 5t | lower int 32t | lower int 5t | Expelled 32t | Expelled 5t |  |
| average | 0 | 0 | 41 | 12 | 10 | 19 | 12 | 1 | 37 | 68 |  |

**Movie S1 (Mutant) Legend:**

Playback at 5 frames per second, each frame represents 1 z-stack capture per minute. This movie shows the abnormal mutant peristaltic process in a 7dpf *shank3abΔC^+/-^* zebrafish. The fluorescent microspheres can be seen near the intestinal bulb-upper intestine junction; this was a common catch point and the mutant model seems to have the greatest difficultly moving food particles out of the intestinal bulb. Post processing was done to reduce size, change orientation of images, and speed playback up.

**Movie S2 (WT) Legend:**

Playback at 5 frames per second, each frame represents 1 z-stack capture per minute. This movie shows “normal” wild type peristaltic movements of a 7dpf zebrafish, highlighting the intestinal region starting at the intestinal bulb-upper intestine junction. Fluorescent microspheres can be seen moving posteriorly down the digestive tract. Post processing was done to reduce size, change orientation of images, and speed playback up.

**Supplemental Materials and Methods:**

*Histology*

Zebrafish clutches were processed en mass at 7dpf to paraffin blocks by fixing in Bouin's fixative for 4 hours at 25^o^C, dehydrating with a graded ethanol series, clearing with xylenes twice for 30 min each, and infiltrating with melted paraffin 2x 30min each. Larval fish were oriented on the sagittal plane in a metal mold and covered with fresh paraffin. Blocks were sectioned at a thickness of 5 µm using a Leitz 1512 microtome after cooling on ice for 15 min, to improve section quality. Due to the young age of the larvae, no deossification was necessary. Sections were deparaffinized with xylenes, rehydrated with a graded ethanol series, and stained, before being dehydrated and mounted in permount (Thermo Fisher; Waltham, MA; UN1294). Sections were stained with Alcian Blue (Alfa Aesar; Haverhill, MA; J60122) and counterstain Eosin B (Alfa Aesar, A17377). Goblet cell counts were acquired through FIJI cell counter; to maintain consistent counts in larval fish, only sections with a clear luminal space that spanned the intestinal bulb all the way to the anus were used, and counts were made using the entirety of the DT length. Adult tissue (supplemental figure 2) was dissected and placed in Bouin's fixative overnight at 25^o^C before processing to paraffin as described above. Sections through adult gut tissue were kept consistent by obtaining transverse sections near the anterior intestine/intestinal bulb transition zone.

*Adult histology*

To acquire adequate CNS tissue for western analysis, several homozygous *shank3abΔC-/-* and WT adult fish (>1yo) were sacrificed and the remaining DT tissue was processed through paraffin, sectioned near the IB and UI transition zone, and stained similar to 7dpf larvae (Fig 5). Adult intestinal lumen (supplemental Fig 2) is similarly well-defined with dense cytoplasm, goblet cells (black arrowheads), and plicae. This intestinal folding has progressed from larval stage to the point where the luminal space is almost completely occluded by the plicae. Goblet cell counts in adult tissue point to significant increases in *shank3abΔC+/-* and *shank3abΔC-/-* mutants as compared to WT (p < 0.001, n=6 for WT, n=6 for *shank3abΔC+/-* and n=6 for *shank3abΔC-/-*).

*Statistics*

For all experiments, WT and *shank3abΔC* ^+/-^ mutant larvae were selected at random from their clutch; multiple clutches were used to avoid any possible batch effect. Goblet and ENS cell counts, and differences in peristaltic periodicity were compared using a one-way ANOVA with a Tukey’s HSD. Due to the non-normal distribution of the microsphere transit data, we used the non-parametric permutation test. To run permutations, we used a custom R script (supplemental table 8) that randomly assigned individual bead occupancy in space and time for the three different treatment groups; WT, unrescued *shank3abΔC*, and rescued *shank3abΔC*. The script ran permutations 5000 times so that we could compare permuted F statistics with the original repeated-measures ANOVA, allowing us to assign p-values. Data for the microsphere transit was also simplified to compare time to complete microsphere expulsion (analogous to completed digestion), as well as intestinal bulb content per timepoint (analogous to gastric emptying). Alpha level cutoff for significance for all tests was set at p < 0.05, and data listing bead transit can be found in Supplementary Tables 3-6. Data listing peristaltic periodicity in the upper and lower intestine can be found in Supplementary Table 7.

*IgorPro code function*

(1) The first image is loaded and displayed, and the user draws a series of line segments along the boundary of each side of the gut (two paths total), and chooses a specific number of boxes along those paths and the height of those boxes (as seen in Figure 3a).  These boxes stay fixed for the entire movie analysis, and for every frame of the movie each box includes the same number of pixels of varying brightness.

(2) To determine the “signal” in each box, the program uses the pixels in each box from the first frame of the movie as the “background” for that box.  Then for box i at time t, the signal s_i(t) = Sum(image - background), where Sum adds, pixel by pixel, the absolute value of the difference between the current image and the background image. So, if the image within a box has changed compared to the first frame (i.e. the gut wall has moved), s(t) will take on a larger value.

(3) Having found s_i(t) for each box, we then normalize all the signals: s_i,norm(t) = [s_i(t) - Min(s_i(t))] / [Max(s_i(t)) - Min(s_i{t))], where Min and Max refer to the smallest and largest signals seen within box i during the entire movie (not including the first frame, because by definition s_i(t=0) is zero).  This gives us a full set of box signals s_i(t) over the whole movie, with values between 0 and 1.

(4) The program constructs a kymograph (not included in fig3), using the normalized s_i(t) signals from each box.  It also makes a compound graph showing all s_i(t) at once (Figure 3b).

(5) The fast Fourier transform (FFT) is computed for each s_i(t).  s_i(t) —> S_i(f), into frequency space, and the program determines which frequency f_max gives the peak signal value (that is, which frequency is the dominant one in the s(t) function), excluding the lowest three possible frequencies because they carry the unwanted dc component of the signal, and not the time-varying part.

(6) The period of the gut motion T_i is determined for each box by inverted f_max: T_i = 1/f_i,max.  So each box is given a period of the motion occurring inside it during the movie.  By averaging all the T_i values, aided by the user visually inspecting the s(t) graphs, the period of gut motion is extracted.
